# Butenolides from the Coral-Derived Fungus *Aspergillius terreus* SCSIO41404

**DOI:** 10.3390/md20030212

**Published:** 2022-03-17

**Authors:** Qingyun Peng, Weihao Chen, Xiuping Lin, Jiao Xiao, Yonghong Liu, Xuefeng Zhou

**Affiliations:** 1 Southern Marine Science and Engineering Guangdong Laboratory (Guangzhou), Guangzhou 511458, China; pengqingyun18@mails.ucas.ac.cn (Q.P.); chenweihao17@mails.ucas.ac.cn (W.C.); yonghongliu@scsio.ac.cn (Y.L.); 2 Research Center for Deepsea Bioresources, Sanya 572025, China; 3 CAS Key Laboratory of Tropical Marine Bio-Resources and Ecology/Guangdong Key Laboratory of Marine Materia Medica, South China Sea Institute of Oceanology, Chinese Academy of Sciences, Guangzhou 510301, China; xiupinglin@hotmail.com; 4 Wuya College of Innovation, Shenyang Pharmaceutical University, Shenyang 110016, China; xj110121@126.com

**Keywords:** butenolides, enantiomers, *Aspergillus terreus*, pancreatic lipase

## Abstract

Five undescribed butenolides including two pairs of enantiomers, (+)-asperteretal G (**1a**), (−)-asperteretal G (**1b**), (+)-asperteretal H (**2a**), (−)-asperteretal H (**2b**), asperteretal I (**3**), and *para*-hydroxybenzaldehyde derivative, (*S*)-3-(2,3-dihydroxy-3-methylbutyl)-4-hydroxybenzaldehyde (**14**), were isolated together with ten previously reported butenolides **4**–**13**, from the coral-derived fungus *Aspergillus terreus* SCSIO41404. Enantiomers **1a**/**1b** and **2a**/**2b** were successfully purified by high performance liquid chromatography (HPLC) using a chiral column, and the enantiomers **1a** and **1b** were new natural products. Structures of the unreported compounds, including the absolute configurations, were elucidated by NMR and MS data, optical rotation, experimental and calculated electronic circular dichroism, induced circular dichroism, and X-ray crystal data. The isolated butenolides were evaluated for antibacterial, cytotoxic, and enzyme inhibitory activities. Compounds **7** and **12** displayed weak antibacterial activity, against *Enterococcus faecalis* (IC_50_ = 25 μg/mL) and *Klebsiella pneumoniae* (IC_50_ = 50 μg/mL), respectively, whereas **6** showed weak inhibitory effect on acetylcholinesterase. Nevertheless, most of the butenolides showed inhibition against pancreatic lipase (PL) with an inhibition rate of 21.2–73.0% at a concentration of 50 μg/mL.

## 1. Introduction

**Figure 1:**
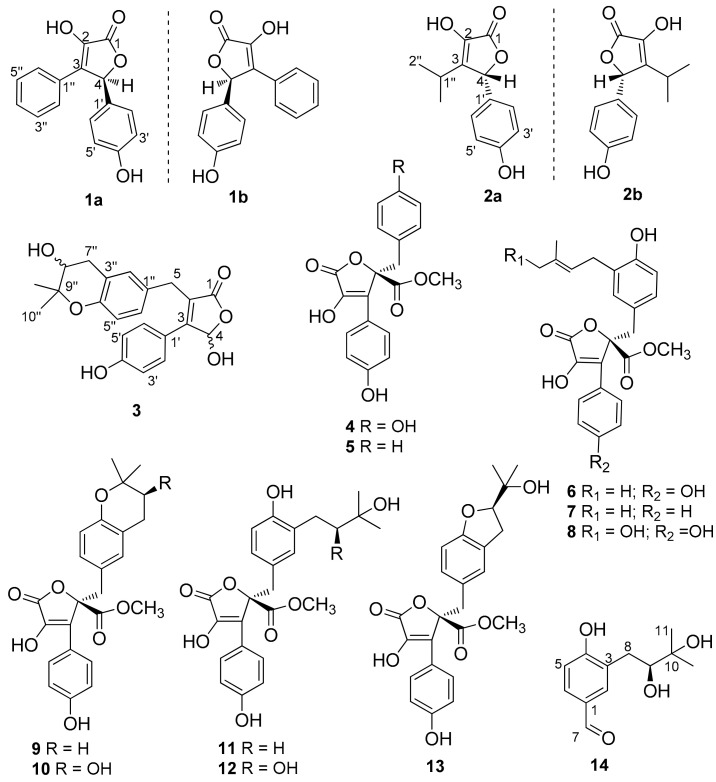
Structures of (**1**–**14**).

Marine-derived fungi have been proven to be a valuable and rich source of novel and bioactive natural products [1]. Butenolides or butyrolactones, possessing the *α*,*β*-unsaturated *γ*-butyrolactone skeleton, were frequently isolated from fungi, especially the *Aspergillus* species [2]. Butenolides have been reported to show diverse biological activities, including anti-inflammatory, cytotoxic, antiviral, antioxidant, antimicrobial, antidiabetic, protein kinase-inhibitory, and *α*-glucosidase-inhibitory activities [2,3,4]. Furthermore, in vivo pharmacological, pharmacokinetics, and metabolism studies have been recently reported [3,5,6], indicating that they could be potential drug leads. Butenolides have also been reported as mycotoxins, possessing significant toxicity such as hepatic and renal oxidative damage [7]. For naturally occurring butenolides, racemic or scalemic mixtures are common [4]. Therefore, separation of racemic or scalemic mixtures to obtain enantiopure compounds is a very difficult task in natural products research. For a discovery of bioactive secondary metabolites, the coral-derived fungus *Aspergillus terreus* SCSIO41404 was investigated. Consequently, five undescribed butenolides (**1a**, **1b**, **2a**, **2b**, and **3**), ten previously reported compounds (**4**–**13**), and an undescribed *para*-hydroxybenzaldehyde derivative (**14**) were isolated and identified from the ethyl acetate (EtOAc) extract of the fungal fermentation (Figure 1). Mixtures of **1a/1b** and **2a/2b** were successfully separated by HPLC using a chiral column to afford two pairs of enantiomers. The first pair of enantiomers (**1a** and **1b**) are considered to be new natural products, since the mixture **1a/1b** has previously been obtained synthetically [8]. Herein, we report the isolation, structure determination, and preliminary antibacterial, cytotoxic, and enzyme inhibitory activity assays of these compounds.

## 2. Results and Discussion

The wheat culture of *Aspergillus* sp. SCSIO41404 was extracted with EtOAc. Several chromatographic methods, including silica gel column and semi-preparative HPLC with octadecylsilyl (ODS) column, were used for isolation of the 16 compounds (Figure 1).

**Figure 2:**
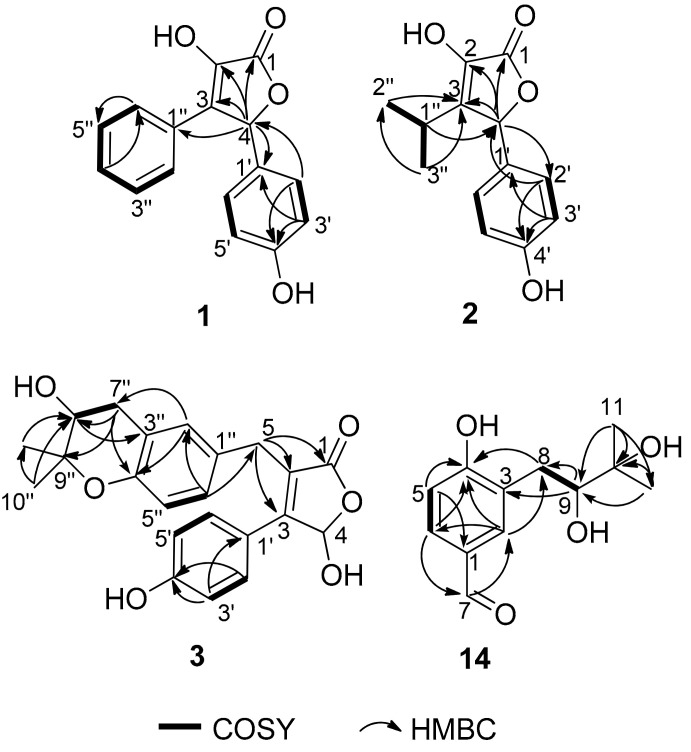
Key COSY and HMBC correlations in (**1**–**3**) and (**14**).

Compound **1** was isolated as a yellow powder and its molecular formula C_16_H_12_O_4_ was based on HRESIMS *m*/*z* 269.0811 [M + H]^+^ (calcd for C_16_H_13_O_4_, 269.0808), accounting for eleven degrees of unsaturation. The ^1^H NMR spectrum (Table 1) showed an A_2_B_2_ spin system of the *para*-disubstituted benzene ring [*δ*_H_ 7.17 (2H, d, *J* = 8.6 Hz, H-2′, H-6′) and 6.74 (2H, d, *J* = 8.6 Hz, H-3′, H-5′)], a monosubstituted benzene ring [*δ*_H_ 7.63 (2H, d, *J* = 7.4 Hz, H-2″, H-6″), 7.28 (2H, t, *J* = 7.4 Hz, H-3″, H-5″) and 7.23 (1H, t, *J* = 7.4 Hz, H-4″)], and a singlet at *δ*_H_ 6.26 (1H, H-4). Besides the above six corresponding hydrogen-bearing carbon signals [*δ*_C_ 130.6 (C-2′/6′), 116.7 (C-3′/5′), 128.9 (C-2″/6″), 129.3 (C-3″/5″), 129.4 (C-4″), 82.4 (C-4)], six carbons remained in the ^13^C NMR spectrum, including an ester carbonyl [*δ*_C_ 171.5 (C-1)] and five non-protonated sp^2^ carbons [*δ*_C_ 140.2 (C-2), 132.2 (C-3), 128.4 (C-1′), 159.7 (C-4′), and 129.5 (C-1″)]. The carbon signals (*δ*_C_ 171.5, 140.2, 132.2, 82.4) and the HMBC correlations from H-4 to C-1, C-2 and C-3, indicated the presence of the butenolide skeleton (Figure 2) [9]. These data showed a close similarity to a synthetic racemic furanone derivative (3-hydroxy-5-(4-hydroxyphenyl)-4-phenyl-2(5*H*)-furanone, **5d** in the reference) [8]. Moreover, the other key HMBC correlations (Figure 2), from H-4 to C-1′ and C-1″, from H-2′/H-6′ to C-4 (*δ*_C_ 82.4) and C-4′, confirmed a planar structure of **1**, which was assigned the trivial name asperteretal G (**1**).

**Figure 3:**
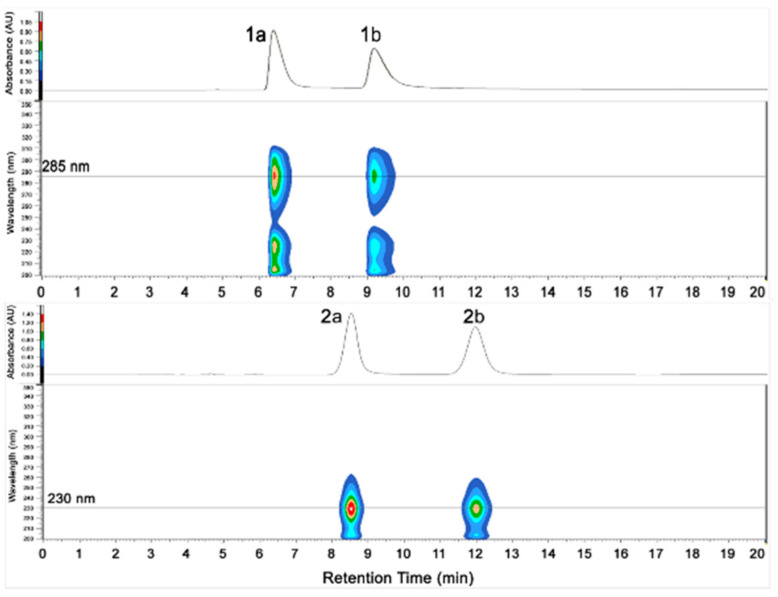
HPLC chromatograms of (±)-**1** and (±)-**2**.

**Figure 4:**
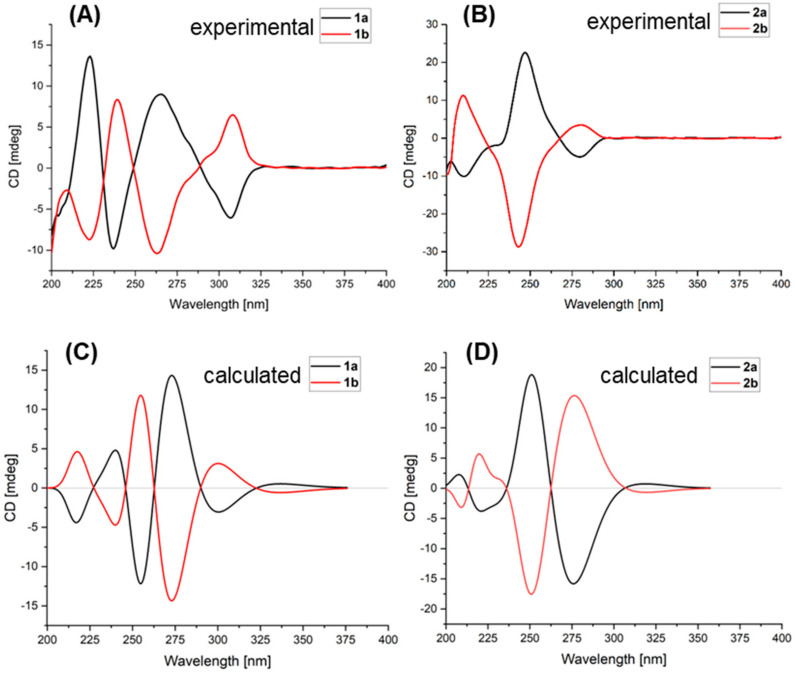
Experimental and calculated ECD spectra of **1a**, **1b** (**A**,**C**), **2a**, and **2b** (**B**,**D**).

**Figure 5:**
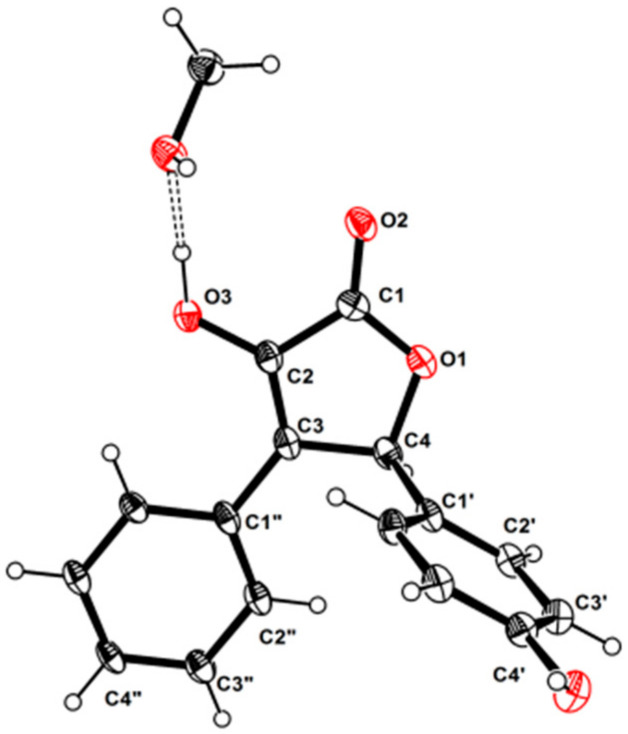
The ortep view of **1a**.

Although **1** has one stereogenic carbon (C-4), its optical rotation was close to zero and the inapparent Cotton effect in the electronic circular dichroism (ECD) spectrum suggested that **1** was not enantiomerically pure. Therefore, **1** was subject to a separation by HPLC using a chiral column CHIRALPAK IC, eluted with isopropanol (IPA)/n-hexane (Hex): 18/82, to obtain two pure enantiomers (**1a** and **1b**, two well-separated peaks in a ratio of approximately 1:1) (Figure 3). The enantiomers **1a** and **1b** possessed opposite Cotton effects in the ECD spectra, and their absolute configurations were determined by comparison of the experimental ECD spectra with those calculated for each enantiomer (Figure 4A,C). The ECD spectra of **1a** and **1b** showed positive and negative Cotton effects in the regions of π→π* transition (260–280 nm), respectively, indicating the 4*S* and 4*R* absolute configurations for **1a** and **1b**, respectively, since **1a** was obtained as a suitable crystal, and its X-ray analysis was performed. The structure of **1a** and the absolute configuration of C-4 were confirmed as shown in the ortep diagram (Figure 5). The crystal data collected on a XtalLAB PRO single-crystal diffractometer using Cu K*α* radiation are in Table S1. Therefore, this pair of enantiomers, as new natural products, were obtained for the first time, and named (+)-asperteretal G (**1a**) and (−)-asperteretal G (**1b**).

Compound **2** was obtained as a colorless oil. Its molecular formula was established as C_13_H_14_O_4_ by HRESIMS *m*/*z* 235.0969 [M + H]^+^ (calcd for C_13_H_15_O_4_, 235.0965), accounting for seven degrees of unsaturation. In the ^1^H NMR spectrum (Table 1) of **2**, the proton signals of a *para*-disubstituted benzene ring [*δ*_H_ 7.09 (2H, d, *J* = 8.6 Hz, H-2′, H-6′) and 6.79 (2H, d, *J* = 8.6 Hz, H-3′, H-5′)] and an isopropyl group [2.49 (1H, hept, *J* = 7.0 Hz, H-1″), 0.99 (3H, d, *J* = 7.0 Hz, H-2″), and 1.08 (3H, d, *J* = 7.0 Hz, H-3″)] were clearly observed. Analysis of the NMR data (Table 1) indicated that **2** had a similar skeleton to that of **1**, except for the phenyl group attached to C-3 in **1** was replaced by an isopropyl group, which was confirmed by HMBC correlations from H_3_-2″ and H_3_-3″ to C-3 (Figure 2). HMBC correlations from H-4 (*δ*_H_ 5.72, s) to C-1 (*δ*_C_ 172.5), C-3 (*δ*_C_ 141.0) and C-2 (*δ*_C_ 138.5), from H-2′/H-6′ to C-4 (*δ*_C_ 83.0) and C-4′ (*δ*_C_ 159.7), and COSY correlations from H-1″ to H_3_-2″, H-1″ and H_3_-3″, confirmed the structure of **2**. Therefore, the planar structure of **2**, named asperteretal H (**2**), was elucidated as shown.

Similarly, the optical rotation and the inapparent ECD Cotton effect also suggested that **2** was not enantiomerically pure. The enantiomers **2a** and **2b** were also successfully separated by HPLC with a chiral column CHIRALPAK IC, eluted with IPA/Hex: 12/88 (two well-separated peaks in a ratio of approximately 1:1) (Figure 3). Enantiomers **2a** and **2b** showed opposite Cotton effects in the ECD spectra, and their absolute configurations were also determined by comparison of the experimental with the calculated ECD spectra for each enantiomer (Figure 4B,D). The positive and negative Cotton effects of **2a** and **2b** in the region near 250 nm, respectively, supporting the 4*R* and 4*S* absolute configurations for **2a** and **2b**, respectively [10,11]. Thus, this pair of enantiomers were identified as (+)-asperteretal H (**2a**) and (−)-asperteretal H (**2b**).

Compound **3** was obtained as a yellow oil. Its molecular formula C_22_H_22_O_6_ was determined based on HRESIMS *m*/*z* 381.1340 [M − H]^−^ (calcd for C_22_H_2__1_O_6_, 381.1344), accounting for twelve degrees of unsaturation. The ^1^H NMR spectrum (Table 1) revealed diagnostic signals of a *para*-disubstituted [*δ*_H_ 7.48 (2H, d, *J* = 8.8 Hz, H-2′, H-6′) and 6.83 (2H, d, *J* = 8.8 Hz, H-3′, H-5′)] and a 1,3,4-trisubstituted benzene rings [*δ*_H_ 6.92 (d, *J* = 2.3 Hz, H-2″), 6.66 (d, *J* = 8.4 Hz, H-5″) and 6.95 (dd, *J* = 8.4, 2.3 Hz, H-6″)], two oxygen-bearing methines [*δ*_H_ 6.48 (s, H-4), 3.72 (H-8″)], two methylenes [*δ*_H_ 3.80 (d, *J* = 15.7 Hz H-5a), 3.72 (H-5b), 2.95 (dd, *J* = 16.6, 4.8 Hz, H-7″a) and 2.66 (dd, *J* = 16.6, 7.3 Hz, H-7″b)], and two methyls [*δ*_H_ 1.30 (3H, s, H-10″), 1.23 (3H, s, H-11″)]. The ^13^C NMR spectrum (Table 1), in combination with DEPT and HSQC spectra, revealed the presence of 20 carbon resonances including two methyls [*δ*_C_ 25.8 (C-10″), 21.2 (C-11″)], two sp^3^ methylenes [*δ*_C_ 32.2 (C-7″), 30.0 (C-5)], eight protonated sp^2^ (*δ*_C_ 131.7, 131.7, 130.3, 128.2, 118.1, 116.6, 116.6, 99.2), one sp^3^ oxygenated methine (*δ*_C_ 70.5) and one ester carbonyl (*δ*_C_ 175.1), seven on-protonated sp^2^ (*δ*_C_ 160.9, 153.0, 158.5, 130.6, 125.7, 123.3, 121.4), and one sp^3^ oxygenated methine (*δ*_C_ 78.0). Its 1D-NMR data were similar to those of asperteretal E [4], except that H_2_-8″ of asperteretal E was replaced by a hydroxyl group (C-8″, *δ*_H/C_ 3.72/70.5). COSY correlation from H_2_-7″ to H-8″ and HMBC correlations from H-8″ to C-3″ and H_2_-7″, H_3_-10″, H_3_-11″ to C-8″ confirmed a planar structure of **3**. Compound **3** was named asperteretal I. Since there are two stereogenic carbons (C-4 and C-8″), there are four possible stereomers for **3**. The inapparent ECD Cotton effect (Figure S1) spectra suggested that **3** was not enantiomerically pure [4]. Our effort to separate the isomers of **3** with various chiral columns and mobile phase systems was not successful. Therefore, the absolute configurations of C-4 and C-8″ in **3** were still undetermined.

**Figure 6:**
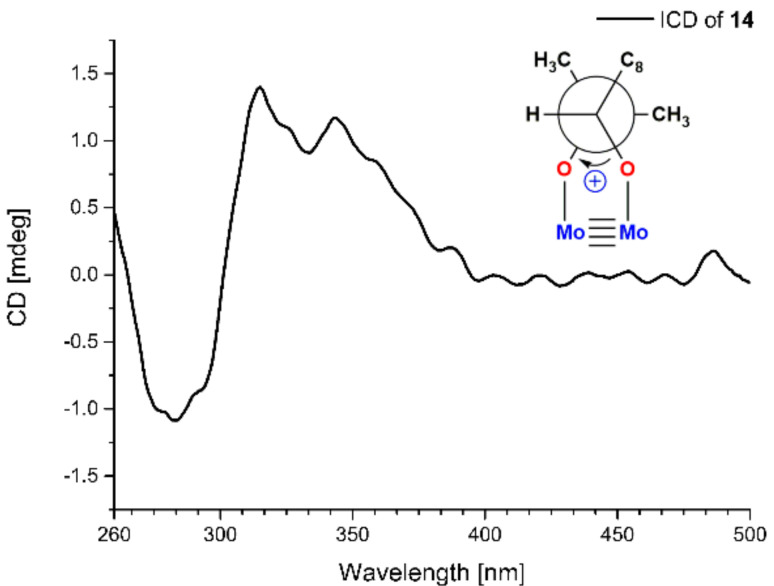
Mo_2_(Oac)_4_-induced CD spectrum of **14**.

Compound **14** was isolated as a yellow oil and had a molecular formula C_12_H_16_O_4_ as determined by HRESIMS *m*/*z* 223.0976 [M − H]^−^ (calcd for C_12_H_1__5_O_4_, 223.0976), accounting for five degrees of unsaturation. Analysis of the NMR data indicated that **14** possessed an aldehyde group (*δ*_H_ 9.75, s, *δ*_C_ 193.1, CHO-7), a 1,2,4-trisubstituted benzene ring (*δ*_H_ 7.75, d, *J* = 2.0 Hz, *δ*_C_ 134.8, CH-2; *δ*_H_ 7.64, dd, *J* = 8.3, 2.0 Hz, *δ*_C_ 131.5, CH-6; *δ*_H_ 6.91, d, *J* = 8.3 Hz, *δ*_C_ 116.7, CH-5), an oxygen-bearing sp^3^ methine (*δ*_H_ 3.63, dd, *J* = 10.4, 1.8 Hz, *δ*_C_ 79.4, CH-9), a sp^3^ methylene (*δ*_H_ 3.07, dd, *J* = 14.1, 1.8 Hz, 2.60, dd, *J* = 14.1, 10.4 Hz, *δ*_C_ 33.8, CH_2_-8), and two methyls (*δ*_H_ 1.25, 6H, s, *δ*_C_ 25.7, 25.1, CH_3_-11 and CH_3_-12). These data were similar to those of 3-(2,3-dihydroxy-isopentyl)-4-hydroxy-acetophenone [12] except for the replacement of the acetyl group on C-1 of 3-(2,3-dihydroxy-isopentyl)-4-hydroxy-acetophenone with an aldehyde group (CHO-7) in **14** [12]. Moreover, the HMBC correlations from H-6 to C-7 and H-7 to C-2 further determined the structure (Figure 1). The absolute configuration of C-9 was determined to be *S* by using the Mo_2_(OAc)_4_–induced circular dichroism method (a positive cotton effect at 314 nm) (Figure 6) [13]. Thus, **14** was identified as (*S*)-3-(2,3-dihydroxy-3-methylbutyl)-4-hydroxybenzaldehyde.

The previously reported compounds were identified as butyrolactone II (**4**) [14], methyl I-2-benzyl-4-hydroxy-3-(4-hydroxyphenyl)-5-oxo-2,5-dihydrofuran-2-carboxylate (**5**) [8,15], butyrolactone I (**6**) [16], versicolactone B (**7**) [17], aspernolide D (**8**) [18], aspernolide A (**9**) [19], butyrolacton V (**10**) [16], terrelactone (**11**) [20], butyrolactone VI (**12**) [21], and butyrolactone IV (**13**) [16] by comparison of their spectroscopic data (Supporting Information, Figures S26–S35) with those reported in the literature.

Compounds **1**–**14** were evaluated for their cytotoxic activity against human lung carcinoma (A549) and human hepatocellular carcinoma (HepG2) cell lines, but none showed cytotoxicity at a concentration of 50 μM. Most of the compounds were screened for their enzyme inhibitory effects against pancreatic lipase (PL) and acetylcholinesterase (AChE) in vitro, and also antibacterial activity against five pathogenic bacteria, *Staphylococcus aureus* ATCC 29213, *Enterococcus faecalis* ATCC 29212, *Klebsiella pneumoniae* ATCC 13883, methicillin-resistant *S. aureus* (MRSA, clinical strain), and methicillin-resistant *S. epidermidis* (MRSE, clinical strain) (Table 2). Only **7** and **12** displayed weak antibacterial activity against *E. faecalis* (IC_50_ value of 25 μg/mL) and *K. pneumoniae* (IC_50_ value of 50 μg/mL), respectively. Compound **6** displayed weak inhibitory effect on AChE (35.2%, at 50 μg/mL concentration). Nevertheless, most of the butenolides (**1a**/**1b**, **2a**/**2b**, **3**, **6**, **7**, **10**, **12**, and **13**) showed inhibition against PL with inhibition rate of 21.2–73.0% at a concentration of 50 μg/mL (Table 2).

## 3. Materials and Methods

### 3.1. General Experimental Procedures

UV spectra were measured on a Shimadzu UV-2600 PC spectrophotometer (Shimadzu, Kyoto, Japan). Optical rotations were recorded on a PerkinElmer MPC 500 (Waltham, MA, USA) polarimeter. For ECD spectra, Chirascan circular dichroism spectrometer (Applied Photophysics, Leatherhead Surrey, UK) was used. NMR spectra were acquired by a Bruker Avance spectrometer (Bruker, Billerica, MA, USA) at 700 MHz for ^1^H and 175 MHz for ^13^C. HRESIMS spectra were recorded on a Bruker miXis TOF-QII mass spectrometer (Bruker, Billerica, MA, USA). X-ray diffraction intensity data were measured on an Agilent Xcalibur Nova single-crystal diffractometer (Santa Clara, CA, USA) using Cu K*α* radiation. TLC and column chromatography were performed on plates precoated with silica gel GF_254_ (10–40 μm) and over silica gel (200–300 mesh) (Qingdao Marine Chemical Factory, Qingdao, China), respectively. Spots were detected on TLC (Qingdao Marine Chemical Factory, Qingdao, China) under 254 nm UV light. Semi-preparative HPLC was performed using an ODS column (YMC-pack ODS-A, YMC Co., Ltd., Kyoto, Japan, 10 mm × 250 mm, 5 μm).

### 3.2. Fungal Material

The fungal strain *Aspergillus* sp. SCSIO41404 was isolated from a soft coral *Sinularia* sp., collected in the Luhuitou waters (109°29′37.3″ E, 18°11′33.4″ N) of Sanya Bay in the South China Sea, in June 2019. The strain was stored on Muller Hinton broth (MB) agar (malt extract 15 g, sea salt 10 g, agar 15 g, H_2_O 1 L, pH 7.4–7.8) at 4 °C and deposited in the CAS Key Laboratory of Tropical Marine Bio-resources and Ecology, South China Sea Institute of Oceanology, Chinese Academy of Sciences, Guangzhou, China. The strain was identified as *Aspergillus terreus* based on the ITS region of the rDNA (GenBank accession No. KU866665.1) (Table S2).

### 3.3. Fermentation and Extraction

The seed medium (malt extract 15 g, sea salt 10 g, H_2_O 1 L, pH 7.4–7.8) in 500 mL Erlenmeyer flasks (150 mL/flask) was incubated at 28 °C for 3 days on a rotating shaker (180 rpm). The seed medium was added to the wheat fermentation medium (wheat 200 g, sea salt 10g, H_2_O 200 mL) in a 1000 mL Erlenmeyer flask. In total, 40 Erlenmeyer flasks were incubated for 30 days at 25 °C without shaking. The whole wheat cultures were crushed and extracted with EtOAc three times to afford an organic extract.

### 3.4. Isolation and Purification

The EtOAc extract (638.1 g) was subjected to silica gel column chromatography eluted with PE-EtOAc-MeOH (50:1:0 to 0:0:1, *v*/*v*) in gradient to yield seven fractions (Frs.1–7). Fr.1 was subjected to HPLC with an ODS column, eluted with MeOH/H_2_O (5–100%) to afford six subfractions (Frs.1-1–1-6). Fr.1-4 was purified by semipreparative HPLC (47% MeOH/H_2_O, 2 mL/min) to yield **5** (4.8 mg). Fr.2 was subjected to MPLC with an ODS column, eluted with MeOH/H_2_O (10–100%) to afford eight subfractions (Frs.2-1–2-8). Fr.2-3 was purified by semipreparative HPLC (60% MeOH/H_2_O, 2 mL/min) to yield **1** (5.9 mg) and **2** (8.5 mg). Fr.3 was purified by semipreparative HPLC (65% MeCN/H_2_O, 2 mL/min) to yield **7** (10.2 mg). Fr.4 gave predominantly **6** (25 g). Fr.5 was purified by semipreparative HPLC (65% MeOH/H_2_O, 2 mL/min) to give **4** (8.9 mg), **9** (3.2 mg), **10** (17.3 mg), and **13** (5.3 mg). Fr.6 was subjected to MPLC with an ODS column, eluting with MeOH/H_2_O (5%100%) to give eight subfractions (Frs.6-1–6-8). Fr.6-4 was purified by semipreparative HPLC (42% MeOH/H_2_O, 2 mL/min) to yield **14** (2.5 mg). Fr.6-6 was purified by semipreparative HPLC (29% MeCN/H_2_O, 2 mL/min) to yield **3** (1.6 mg), **8** (0.81 mg), **11** (5.4 mg), and **12** (10.2 mg). 

*Asperteretal G* (**1**): yellow powder; UV (MeOH) *λ*_max_ (log *ε*) = 203 (4.37), 221 (4.21), 226 (4.20), 286 (4.17), 327 (3.80) nm; ^1^H and ^13^C NMR data, Table 1; HRESIMS *m*/*z* 269.0811 [M + H]^+^ (calcd for C_16_H_13_O_4_, 269.0808) (Figures S2–S9).

*(+)-asperteretal G* (**1a**): $[\alpha {]}_{D}^{25}$ +36.9 (*c* 0.1, MeOH); ECD (0.2 mg/mL, IPA) *λ*_max_ (Δ*ε*) 223 (+5.56), 237 (−4.04), 265 (+3.67), 307 (−2.51) nm.

*(−)-asperteretal G* (**1b**): $[\alpha {]}_{D}^{25}$ −23.7 (*c* 0.1, MeOH); ECD (0.2 mg/mL, IPA) *λ*_max_ (Δ*ε*) 223 (−3.57), 239 (+3.40), 263 (−4.23), 308 (+2.64) nm.

*Asperteretal H* (**2**): colorless oil; UV (MeOH) *λ*_max_ (log *ε*) = 202 (4.28), 230 (4.33), 277 (3.53), 283 (3.51), 310 (3.42) nm; ^1^H and ^13^C NMR data, Table 1; HRESIMS *m*/*z* 235.0969 [M + H]^+^ (calcd for C_13_H_15_O_4_, 235.0965) (Figures S10–S17).

*(+)-asperteretal H* (**2a**): $[\alpha {]}_{D}^{25}$ +68.2 (*c* 0.1, MeOH); ECD (0.2 mg/mL, IPA) *λ*_max_ (Δ*ε*) 210 (−3.59), 247 (+8.08), 280 (−1.76) nm.

*(−)-asperteretal H* (**2b**): $[\alpha {]}_{D}^{25}$ −39.7 (*c* 0.1, MeOH); ECD (0.2 mg/mL, IPA) *λ*_max_ (Δ*ε*) 210 (+4.10), 243 (−10.27), 281 (+1.24) nm.

*Asperteretal I* (**3**): yellow oil;$[\alpha {]}_{D}^{25}$ +8.0 (*c* 0.1, MeOH); UV (MeOH) *λ*_max_ (log *ε*) = 203 (4.45), 305 (4.09) nm; ^1^H and ^13^C NMR data, Table 1; HRESIMS *m*/*z* 381.1340 [M − H]^−^ (calcd for C_22_H_2__1_O_6_, 381.1344) (Figures S18–S25).

*(S)-3-(2,3-Dihydroxy-3-methylbutyl)-4-hydroxybenzaldehyde* (**14**): yellow oil; $[\alpha {]}_{D}^{25}$ +7.7 (*c* 0.10, MeOH); UV (MeOH) *λ*_max_ (log *ε*) = 203 (3.88), 224 (3.49), 276 (3.13) nm; ^1^H NMR (700 MHz, CD_3_OD) *δ*_H_ 9.75 (1H, s, H-7), 7.75 (1H, d, *J* = 2.0 Hz, H-2), 7.64 (1H, dd, *J* = 8.3, 2.0 Hz, H-6), 6.91 (1H, d, *J* = 8.3 Hz, H-5), 3.63 (1H, dd, *J* = 10.4, 1.8 Hz, H-9), 3.07 (1H, dd, *J* = 14.1, 1.8 Hz, H_a_-8), 2.60 (1H, dd, *J* = 14.1, 10.4 Hz, H_b_-8), 1.25 (6H, s, H-11, H-12); ^13^C NMR (175 MHz, CD_3_OD) *δ*_C_ 193.1 (CH, C-7), 164.0 (qC, C-4), 134.8 (CH, C-2), 131.5 (CH, C-6), 130.1 (qC, C-1), 129.1 (qC, C-3), 116.7 (CH, C-5), 79.4 (CH, C-9), 73.9 (qC, C-10), 33.8 (CH_2_, C-8), 25.7 (CH_3_, C-12), 25.1 (CH_3_, C-11); HRESIMS *m*/*z* 223.0976 [M − H]^−^ (calcd for C_12_H_1__5_O_4_, 223.0976) (Figures S36–S43).

### 3.5. X-ray Crystallographic Analysis

Colorless crystals of **1a** were obtained in MeOH/CHCl_3_ (1:2) followed by slow evaporation at 4 °C and the crystals’ data were collected on a XtalLAB PRO single-crystal diffractometer using Cu K*α* radiation. The X-ray crystal structure of **1a** was solved using SHELXS97, expanded by difference Fourier techniques, and refined by full-matrix least-squares calculation finally. All non-hydrogen atoms were refined anisotropically, and hydrogen atoms were fixed at calculated positions. Crystallographic data of **1a** (Table S1) have been deposited in the Cambridge Crystallographic Data Centre (deposition number: CCDC 2077455). These data can be obtained, free of charge, on application to CCDC, 12 Union Road, Cambridge CB21EZ, UK [fax: +44(0)-1223-336033 or e-mail: deposit@ccdc.cam.ac.uk].

### 3.6. Mo_2_(AcO)_4_-Induced Circular Dichroism

Mo_2_(AcO)_4_ (1.0 mg) and **14** (1.0 mg) were dissolved in dimethyl sulfoxide (DMSO) (1 mL) to use as stock solutions, which were mixed by 1:2 *v*/*v*. After mixing, the CD spectrum was recorded immediately and scanned every 5 min, until a stationary ICD spectrum (the CD of **14** in Mo_2_(AcO)_4_ solution subtracted from inherent CD of **14** in DMSO) was observed. The sign of the diagnostic band at around 310 nm in the ICD spectrum was correlated to the absolute configuration of C-9 of **14** [22].

### 3.7. ECD Calculation

The structures of **1a**, **1b**, **2a**, and **2b** were subjected to random conformational searches using the Spartan’14 software with the MMFF method, as used previously [23]. The conformers with a Boltzmann population of over 5% were chosen for ECD calculations using the Gaussian 09 software [24], and the stable conformers were initially optimized at the B3LYP/6-31+G(d,p) level in MeOH using the CPCM model. The overall theoretical calculation of ECD was achieved in MeOH using time-dependent density functional theory at the B3LYP/6-31+G (d, p) level. The ECD spectra were generated using the SpecDis 1.6 (University of Würzburg, Würzburg, Germany) and Prism 5.0 (GraphPad Software Inc., San Diego, CA, USA) software with a half-bandwidth of 0.3–0.4 eV, according to the Boltzmann-calculated contribution of each conformer after UV correction.

### 3.8. Bioassay

The cytotoxic activity of the obtained compounds was evaluated by the MTT method as reported in our previous study [25]. The antibacterial activity against five bacterial strains, *Staphylococcus aureus* ATCC 29213, *Enterococcus faecalis* ATCC 29212, *Klebsiella pneumoniae* ATCC 13883, methicillin-resistant *S. aureus* (MRSA, clinical strain), and methicillin-resistant *S. epidermidis* (MRSE, clinical strain) was evaluated using a modified broth microdilution method [25,26]. The acetylcholinesterase and PL inhibitory activities were evaluated according to the methods used in our previous study [26,27].

## 4. Conclusions

We describe here the isolation and structure elucidation of five undescribed and ten previously reported butenolides from the coral-derived fungus *Aspergillus terreus* SCSIO41404, together with an undescribed *para*-hydroxybenzaldehyde derivative (*S*)-3-(2,3-dihydroxy-3-methylbutyl)-4-hydroxybenzaldehyde. Two pairs of enantiomers were separated by HPLC using a chiral column, and enantiomers (+)-asperteretal G (**1a**) and (−)-asperteretal G (**1b**) were new natural products. After preliminary antibacterial, cytotoxic and enzyme inhibitory bioassays of these compounds, several natural butenolides showed inhibition against PL. This is the first report of the PL inhibitor activity of butenolides, and the effects of these butenolides on the regulation of lipid metabolism deserve further study [28].

## Figures and Tables

**Table 1:** ^1^H NMR (700 MHz) and ^13^C NMR (175 MHz) Data for **1**–**3** in CD_3_OD.

	1		2		3	
No	*δ*_C_, Type	*δ*_H_ (*J* in Hz)	*δ*_C_, Type	*δ*_H_ (*J* in Hz)	*δ*_C_, Type	*δ*_H_ (*J* in Hz)
1	171.5, C		172.5, C		175.1, C	
2	140.2, C		138.5, C		125.7, C	
3	132.2, C		141.0, C		158.5, C	
4	82.4, CH	6.26, s	83.0, CH	5.72, s	99.2, CH	6.48, s
5					30.0, CH_2_	3.72, overlapped
						3.80, d (15.7)
1′	128.4, C		127.6, C		123.3, C	
2′	130.6, CH	7.17, d (8.6)	130.1, CH	7.09, d (8.6)	131.7, CH	7.48, d (8.8)
3′	116.7, CH	6.74, d (8.6)	116.5, CH	6.79, d (8.6)	116.6, CH	6.83, d (8.8)
4′	159.7, C		159.7, C		160.9, C	
5′	116.7, CH	6.74, d (8.6)	116.5, CH	6.79, d (8.6)	116.6, CH	6.83, d (8.8)
6′	130.6, CH	7.17, d (8.6)	130.1, CH	7.09, d (8.6)	131.7, CH	7.48, d (8.8)
1″	129.5, C		27.4, CH	2.49, hept (7.0)	130.6, C	
2″	128.9, CH	7.63, d (7.4)	21.0, CH_3_	0.99, d (7.0)	130.3, CH	6.92, d (2.3)
3″	129.3, CH	7.28, t (7.4)	20.1, CH_3_	1.08, d (7.0)	121.4, C	
4″	129.4, CH	7.23, t (7,4)			153.0, C	
5″	129.3, CH	7.28, t (7.4)			118.1, CH	6.66, d (8.4)
6″	128.9, CH	7.63, d (7.4)			128.2, CH	6.95, dd (8.4, 2.3)
7″					32.2, CH_2_	2.66, dd (16.6, 7.3)
						2.95, dd (16.6, 4.8)
8″					70.5, CH	3.72, overlapped
9″					78.0, C	
10″					25.8, CH_3_	1.30, s
11″					21.2, CH_3_	1.23, s

**Table 2:** The enzyme inhibitory and antibacterial activities of **1**–**7** and **10**–**13**.

Comp.	Enzyme Inhibition Rate at 50 μg/mL (%)		Antibacterial Activities (MIC, μg/mL)	
	PL	AChE	*E. faecalis*	*K. pneumoniae*
**1a/1b**	58.8	<10	>100	>100
**2a/2b**	67.2	<10	>100	>100
**3**	35.5	<10	>100	>100
**4**	<10	<10	>100	>100
**5**	<10	<10	>100	>100
**6**	37.6	35.2	>100	100
**7**	73.0	<10	25	>100
**10**	54.1	<10	>100	>100
**11**	<10	<10	>100	>100
**12**	21.2	<10	>100	50
**13**	66.8	<10	>100	>100
Control	86.5 ^a^	83.7 ^b^	4 ^c^	0.5 ^c^

^a^ Orlistat, ^b^ tacrine, and ^c^ ampicillin were used as positive controls.

## Data Availability

Not applicable.

## Supplementary Materials

The following supporting information can be downloaded at: https://www.mdpi.com/article/10.3390/md20030212/s1, ITS sequence data of the strain; physicochemical data of **4**–**13**; Table S1: X-ray crystallographic data of **1**; Table S2: Primer sequences for the genes; Figure S1: Experimental ECD spectrum of **3**; Figures S2–S25: NMR, HRESIMS and UV spectroscopic data of **1**–**3**; Figures S26–S35: ^1^H NMR spectra of **4**–**13**; Figures S36–S43: NMR, HRESIMS and UV spectroscopic data of **14** (PDF); Crystallographic data of **1** (CIF).
