# Supplementary material for: Butenolides from the Coral-Derived Fungus Aspergillius terreus SCSIO41404
Source: Mar Drugs. 2022 Mar 17;20(3):212. doi: 10.3390/md20030212 (PMC8955524; doi:10.3390/md20030212)
Supplement: Supplementary file 1 [file marinedrugs-20-00212-s001.zip › marinedrugs-1578321-supplementary.pdf]

Supporting Information

Butenolides from the Coral-Derived Fungus *Aspergillius terreus* SCSIO41404

Qingyun Peng [1,2,†], Weihao Chen [1,3,†], Xiuping Lin [3], Jiao Xiao [4], Yonghong Liu, [1,3,4] and Xuefeng Zhou [1,3,*]

[1] Southern Marine Science and Engineering Guangdong Laboratory (Guangzhou), Guangzhou 511458, China; pengqingyun18@mails.ucas.ac.cn (Q.P.); chenweihao17@mails.ucas.ac.cn (W.C.); yonghongliu@scsio.ac.cn (Y.L.)
[2] Research Center for Deepsea Bioresources, Sanya, Hainan 572025, China;
[3] CAS Key Laboratory of Tropical Marine Bio-resources and Ecology/Guangdong Key Laboratory of Marine Materia Medica, South China Sea Institute of Oceanology, Chinese Academy of Sciences, Guangzhou 510301, China; xiupinglin@hotmail.com (X.L.)
[4] Wuya College of Innovation, Shenyang Pharmaceutical University, Shenyang 110016, China; xj110121@126.com (J.X.)
* Correspondence: xfzhou@scsio.ac.cn (X.Z.)
† These authors contributed equally to this work.

Contents

ITS sequence of the strain *Aspergillus terreus* SCSIO41404……………………..3

Physicochemical data of 4–13. ……………………………………………………..…..3

Table S1. X-ray Crystallographic Data of **1**. ……………………………………..……..7

Table S2. The primer sequences for the genes. ……………………………...……..8

Figure S1. Experimental ECD spectrum of **3**. ………………………………………..8

Figure S2. ^{1}H NMR spectrum of **1** (CD_3OD, 700 MHz). ……………….......………..9

Figure S3. ^{13}C NMR spectrum of **1** (CD_3OD, 175 MHz). ………………..………..9

Figure S4. DEPT spectrum of **1** (CD_3OD). ………………………………………..…..10

Figure S5. COSY spectrum of **1** (CD_3OD). ………………………………………..…..10

Figure S6. HSQC spectrum of **1** (CD_3OD). ………………………………………....11

Figure S7. HMBC spectrum of **1** (CD_3OD). …...………..11

Figure S8. HRESIMS spectrum of **1**. ……………………………………………..…..12

Figure S9. UV spectrum of **1**. ……………………………………………………..12

Figure S10. ^{1}H NMR spectrum of **2** (CD_3OD, 700 MHz). ……………………..…..13

Figure S11. ^{13}C NMR spectrum of **2** (CD_3OD, 175 MHz). ……………………..…..13

Figure S12. DEPT spectrum of **2** (CD_3OD). ……………………………………..…..14

Figure S13. COSY spectrum of **2** (CD_3OD). ……………………………………..…..14

Figure S14. HSQC spectrum of **2** (CD_3OD). ……………………………………..…..15

Figure S15. HMBC spectrum of **2** (CD_3OD). ……………………………………...15

Figure S16. HRESIMS spectrum of **2**. ……………………………………………...16

Figure S17. UV spectrum of **2**. ……………………………………………………...16

Figure S18. ^1H NMR spectrum of **3** (CD_3OD, 700 MHz). ……………….………...17

Figure S19. ^{13}C NMR spectrum of **3** (CD_3OD, 175 MHz). ……………………....17

Figure S20. DEPT spectrum of **3** (CD_3OD). ………………………………………...18

Figure S21. COSY spectrum of **3** (CD_3OD). ………………………………………...18

Figure S22. HSQC spectrum of **3** (CD_3OD). ………………………………...…..19

Figure S23. HMBC spectrum of **3** (CD_3OD). ……………………………………..…..19

Figure S24. HRESIMS spectrum of **3**. ………………………………………….…...20

Figure S25. UV spectrum of **3**. ……………………………………………………...20

Figure S26. ^1H NMR spectrum of **4** (DMSO-d_6, 700 MHz). ……………….…..21

Figure S27. ^1H NMR spectrum of **5** (CD_3OD, 700 MHz). ………………………....21

Figure S28. ^1H NMR spectrum of **6** (CD_3OD, 700 MHz). ……………………..…..22

Figure S29. ^1H NMR spectrum of **7** (CD_3OD, 700 MHz). ……………………..…..22

Figure S30. ^1H NMR spectrum of **8** (CD_3OD, 700 MHz). ………………………....23

Figure S31. ^1H NMR spectrum of **9** (CD_3OD, 700 MHz). ……………………..…..23

Figure S32. ^1H NMR spectrum of **10** ($CDCl_3$, 700 MHz). ………………………....24

Figure S33. ^1H NMR spectrum of **11** (CD_3OD, 700 MHz). ………………….……..24

Figure S34. ^1H NMR spectrum of **12** (CD_3OD, 700 MHz). ……………………...25

Figure S35. ^1H NMR spectrum of **13** (CD_3OD, 700 MHz). ………………….……..25

Figure S36. ^1H NMR spectrum of **14** (CD_3OD, 700 MHz). ……………………...26

Figure S37. ^{13}C NMR spectrum of **14** (CD_3OD, 175 MHz). ……………………..26

Figure S38. DEPT spectrum of **14** (CD_3OD). …………………………………….... 27

Figure S39. COSY spectrum of **14** (CD_3OD). ………………………………….….. 27

Figure S40. HSQC spectrum of **14** (CD_3OD). …………………………………….. 28

Figure S41. HMBC spectrum of **14** (CD_3OD). ……………………………………..28

Figure S42. HRESIMS spectrum of **14**. ……………………………………...……..29

Figure S43. UV spectrum of **14**.……………………………………………………..29

ITS sequence of the strain *Aspergillus terreus* SCSIO41404.

GGTAAAGCGCTGGTCTTATGGCCACCTCCCACCCGTGACTATTGTACCTTGT TGCTTCGGCGGGCCCGCCAGCGTTTGCTGGCCGCCGGGGGGCGACTCGCC CCCGGGCCCGTGCCCGCCGGAGACCCCAACATGAACCCTGTTCTGAAAGC TTGCAGTCTGAGTGTGATTCTTTGCAATCAGTTAAAACTTTCAACAATGGAT CTCTTGGTTCCGGCATCGATGAAGAACGCAGCGAAATGCGATAACTAATGT GAATTGCAGAATTCAGTGAATCATCGAGTCTTTGAACGCACATTGCGCCCC CTGGTATTCCGGGGGGCATGCCTGTCCGAGCGTCATTGCTGCCCTCAAGCC CGGCTTGTGTGTTGGGCCCTCGTCCCCCGGCTCCCGGGGGACGGGCCCGA AAGGCAGCGGCGGCACCGCGTCCGGTCCTCGAGCGTATGGGGCTTCGTCT TCCGCTCCGTAGGCCCGGCCGGCGCCCGCCGACGCATTTATTTGCAACTTG TTTTTTTTTCCAGGTTGACCTCGGATCAGGTAGGGATACCCGCTGAACTTAA GCATATCAATAAGCGGAGGAA

Physicochemical data of 4–13.

Butyrolactone II (**4**): yellow oil; $[\alpha]_D^{20}$ = +30.5 (*c* 0.1, MeOH); ^{1}H NMR (700 MHz, DMSO-d_6) δ_H 10.57 (1H, s, 2-OH), 9.95 (1H, s, 4′-OH), 9.22 (1H, s, 4″-OH), 7.49 (2H, d, *J* = 8.7 Hz, H-2′, H-6′), 6.85 (2H, d, *J* = 8.7 Hz, H-3′, H-5′), 6.56 (2H, d, *J* = 8.5 Hz, H-2″, H-6″), 6.48 (2H, d, *J* = 8.5 Hz, H-3″, H-5″), 3.71 (3H, s, 5-OCH$_3$), 3.37 (2H, d, *J* = 9.7 Hz, H-6); ^{13}C NMR (175 MHz, DMSO-d_6) δ_C 169.8 (qC, C-5), 167.9 (qC, C-1), 157.9 (qC, C-4′), 156.3 (qC, C-4″), 138.1 (qC, C-2), 131.2 (CH, C-2″, C-6″), 128.8 (CH, C-2′, C-6′), 127.5 (qC, C-3), 123.2 (qC, C-1″), 121.0 (qC, C-1′), 115.9 (CH, C-3′, C-5′), 114.6 (CH, C-3″, C-5″), 84.7 (qC, C-4), 53.5 (CH$_3$, 5-OCH$_3$), 38.0 (CH$_2$, C-6).

Methyl (*R*)-2-benzyl-4-hydroxy-3-(4-hydroxyphenyl)-5-oxo-2,5-dihydrofuran-2-carboxylate (**5**): yellow oil; $[\alpha]_D^{25}$ = +65.6 (*c* 0.1, MeOH); ^{1}H NMR (700 MHz, CD$_3$OD) δ_H 7.58 (2H, d, *J* = 8.8 Hz, H-2′, H-6′), 7.13 (1H, t, *J* = 7.3 Hz, H-4″), 7.09 (2H, t, *J* = 7.3 Hz, H-3″, H-5″), 6.87 (2H, d, *J* = 8.8 Hz, H-3′, H-5′), 6.83 (2H d, *J* = 7.3 Hz, H-2″, H-6″), 3.79 (3H, s, 5-OCH$_3$), 3.56 (2H, s, H-6); ^{13}C NMR (175 MHz, CD$_3$OD) δ_C 171.4 (qC, C-5) 170.1 (qC, C-1), 159.4 (qC, C-4′), 139.7 (qC, C-2), 134.7 (qC, C-1″), 131.5 (CH, C-2′, C-6′), 130.3 (CH, C-2″, C-6″), 129.0 (qC, C-3), 128.8 (CH, C-3″, C-5″), 128.1 (CH, C-4″), 123.0 (qC, C-1′), 116.6 (CH, C-3′, C-5′), 86.5 (qC, C-4), 53.9 (CH$_3$, 5-OCH$_3$), 40.2 (CH$_2$, C-6).

Butyrolactone I (**6**): yellow oil; $[\alpha]_D^{20}$ = +64.0 (*c* 0.2, MeOH); ^{1}H NMR (700 MHz, CD$_3$OD) δ_H 7.59 (2H, d, *J* = 8.8 Hz, H-2′, H-6′), 6.87 (2H, d, *J* = 8.8 Hz, H-3′, H-5′), 6.54 (1H, dd, *J* = 8.2, 2.1 Hz, H-6″), 6.50 (1H, d, *J* = 8.2 Hz, H-5″), 6.41 (1H, d,

J = 2.1 Hz, H-2″), 5.06 (1H, m, H-8″), 3.78 (3H, s, 5-OCH_3), 3.45 (1H, d, J = 14.8 Hz, H_a-6), 3.41 (1H, d, J = 14.8 Hz, H_b-6), 3.07 (2H, m, H-7″), 1.67 (3H, br s, H-11″), 1.57 (3H, br s, H-10″); ^{13}C NMR (175 MHz, CD_3OD) δ_C 171.6 (qC, C-5), 170.4 (qC, C-1), 159.3 (qC, C-4′), 155.1 (qC, C-4″), 139.7 (qC, C-2), 133.0 (qC, C-9″), 132.4 (CH, C-2″), 130.4 (CH, C-2′, C-6′), 129.8 (CH, C-6″), 129.1 (qC, C-3), 128.4 (qC, C-3″), 125.1 (qC, C-1″), 123.6 (CH, C-8″), 123.2 (qC, C-1′), 116.6 (CH, C-3′, C-5′), 115.0 (CH, C-5″), 86.8 (qC, C-4), 53.8 (CH_3, 5-OCH_3), 39.6 (CH_2, C-6), 28.7 (CH_2, C-7″), 26.0 (CH_3, C-11), 17.8 (CH_3, C-10″).

Versicolactone B (**7**): white powder; $[\alpha]_D^{25}$ = +67.7 (c 0.1, MeOH);; ^1H NMR (700 MHz, CD_3OD) δ_H 7.70 (2H, d, J = 7.5 Hz, H-2′, H-6′), 7.46 (2H, t, J = 7.5 Hz, H-3′, H-5′), 7.38 (1H, t, J = 7.5 Hz, H-4′), 6.51 (1H, dd, J = 8.2, 2.0 Hz, H-6″), 6.49 (1H, d, J = 8.2 Hz, H-5″), 6.40 (1H, d, J = 2.0 Hz, H-2″), 5.06 (1H, m, H-8″), 3.79 (3H, s, 5-OCH_3), 3.45 (2H, br s, H-6), 3.07 (2H, m, H-7″), 1.65 (3H, br s, H-11″), 1.57 (3H, br s, H-10″); ^{13}C NMR (175 MHz, CD_3OD) δ_C 171.5 (qC, C-5), 169.9 (qC, C-1), 155.2 (qC, C-4″), 141.9 (qC, C-2), 132.9 (qC, C-9″), 132.4 (CH, C-2″), 131.9 (CH, C-2′, C-6′), 129.8 (CH, C-3′, C-5′, C-6″), 129.7 (qC, C-3), 128.6 (CH, C-4′), 128.5 (qC, C-3″), 128.0 (qC, C-1′), 125.0 (qC, C-1″), 123.6 (CH, C-8″), 115.1 (CH, C-5″), 86.9 (qC, C-4), 53.9 (CH_3, 5-OCH_3), 39.5 (CH_2, C-6), 28.8 (CH_2, C-7″), 26.0 (CH_3, C-11), 17.8 (CH_3, C-10″).

Aspernolide D (**8**): white powder; $[\alpha]_D^{25}$ = +61.3 (c 0.1, MeOH); ^1H NMR (700 MHz, CD_3OD) δ_H 7.58 (2H, d, J = 8.7 Hz, H-2′, H-6′), 6.87 (2H, d, J = 8.7 Hz, H-3′, H-5′), 6.50 (1H, dd, J = 8.1, 1.9 Hz, H-6″), 6.48 (1H, d, J = 8.1 Hz, H-5″), 6.47 (1H, d, J = 1.9 Hz, H-2″), 5.39 (1H, m, H-8″), 3.91 (2H, br s, H-10″), 3.78 (3H, s, 5-OCH_3), 3.45 (1H, d, J = 14.6 Hz, H_a-6), 3.41 (1H, d, J = 14.6 Hz, H_b-6), 3.15 (2H, d, J = 7.4 Hz, H-7″), 1.65 (3H, br s, H-11″); ^{13}C NMR (175 MHz, CD_3OD) δ_C 171.6 (qC, C-5), 170.4 (qC, C-1), 159.4 (qC, C-4′), 155.2 (qC, C-4″), 139.8 (qC, C-2), 136.3 (qC, C-9″), 132.7 (CH, C-2″), 130.4 (CH, C-2′, C-6′), 129.9 (CH, C-6″), 129.2 (qC, C-3), 127.9 (qC, C-3″), 125.3 (CH, C-8″), 125.2 (qC, C-1″), 123.2 (qC, C-1′), 116.6 (CH, C-3′, C-5′), 115.1 (CH, C-5″), 86.9 (qC, C-4), 69.1 (CH_2, C-10″), 53.9 (CH_3, 5-OCH_3), 39.5 (CH_2, C-6), 28.6 (CH_2, C-7″), 13.8 (CH_3, C-11).

Aspernolide A (**9**): yellow oil; $[\alpha]_D^{25}$ = +38.6 (c 0.1, MeOH); ^1H NMR (700 MHz, CD_3OD) δ_H 7.56 (2H, d, J = 8.9 Hz, H-2′, H-6′), 6.87 (2H, d, J = 8.9 Hz, H-3′, H-5′), 6.51 (1H, dd, J = 8.3, 2.1 Hz, H-6″), 6.48 (1H, d, J = 2.1 Hz, H-2″), 6.43 (1H, d, J = 8.3 Hz, H-5″), 3.79 (3H, s, 5-OCH_3), 3.44 (2H, s, H-6), 2.58 (2H, m, H-7″), 1.72 (2H,

t, J = 6.8 Hz, H-8″), 1.25(3H, s, H-11″), 1.24 (3H, s, H-10″); ^{13}C NMR (175 MHz, CD_3OD) δ_C 171.5 (qC, C-5), 170.3 (qC, C-1), 159.4 (qC, C-4′), 154.3 (qC, C-4″), 139.7 (qC, C-2), 132.6 (CH C-2″), 130.4 (CH, C-2′, C-6′), 130.2 (CH, C-6″), 129.3 (qC, C-3), 125.5 (qC, C-3″), 123.1 (qC, C-1″), 121.4 (qC, C-1′), 117.4 (CH, C-5″), 116.6 (CH, C-3′, C-5′), 86.8 (qC, C-4), 75.1 (qC, C-9″), 53.9 (CH_3, 5-OCH_3), 39.6 (CH_2, C-6), 33.7 (CH_2, C-8″), 27.0 (CH_3, C-10″), 26.9 (CH_3, C-11″), 23.2 (CH_2, C-7″).

Butyrolactone V (**10**): yellow oil; $[\alpha]_D^{20}$ = +70.0 (*c* 0.1, MeOH); ^{1}H NMR (700 MHz, $CDCl_3$) δ_H 7.59 (2H, d, J = 8.6 Hz, H-2′, H-6′), 6.90 (2H, d, J = 8.6 Hz, H-3′, H-5′), 6.59 (1H, br s, H-2″), 6.54 (2H, overlapped, H-5″, H-6″), 3.78 (3H, s, 5-OCH_3), 3.76 (1H. t, J = 4.9 Hz, H-8″), 3.54 (1H, d, J = 14.8 Hz, H_a-6), 3. 45 (1H, d, J = 14.8 Hz, H_b-6), 2.87 (1H, dd, J = 16.8, 4.9 Hz, H_a-7″), 2.62 (1H, dd, J = 16.8, 4.9 Hz, H_b-7″), 1.31(3H, s, H-10″), 1.24 (3H, s, H-11″); ^{13}C NMR (175 MHz, $CDCl_3$) δ_C 169.9 (qC, C-5), 169.4 (qC, C-1), 156.7 (qC, C-4′), 152.1 (qC, C-4″), 137.5 (qC, C-2), 132.2 (CH C-2″), 129.8 (CH, C-6″), 129.7 (CH, C-2′, C-6′), 128.3 (qC, C-3), 124.9 (qC, C-1″), 122.4 (qC, C-1′), 118.3 (qC, C-3″), 116.9 (CH, C-5″), 116.2 (CH, C-3′, C-5′), 86.1 (qC, C-4), 76.9 (qC, C-9″), 69.8 (CH, C-8″), 53.7 (CH_3, 5-OCH_3), 38.8 (CH_2, C-6), 31.2 (CH_2, C-7″), 24.8 (CH_3, C-10″), 22.5 (CH_3, C-11″).

Terrelactone (**11**): yellow oil; $[\alpha]_D^{25}$ = +54.1 (*c* 0.1, MeOH); ESIMS *m/z* 465.5 [M + Na]$^+$; ^{1}H NMR (700 MHz, CD_3OD) δ_H 7.58 (2H, d, J = 8.8 Hz, H-2′, H-6′), 6.87 (2H, d, J = 8.8 Hz, H-3′, H-5′), 6.50 (1H, dd, J = 8.1, 2.1 Hz, H-6″), 6.48 (1H, d, J = 8.1 Hz, H-5″), 6.45 (1H, d, J = 2.1 Hz, H-2″), 3.79 (3H, s, 5-OCH_3), 3.45 (1H, d, J = 14.7 Hz, H_a-6), 3.41 (1H, d, J = 14.7 Hz, H_b-6), 2.49 (1H, m, H_a-7″), 2.40 (1H, m, H_b-7″), 1.53 (2H, m, H-8″), 1.20 (3H, s, H-10″), 1.19 (3H, s, H-11″); ^{13}C NMR (175 MHz, CD_3OD) δ_C 171.6 (qC, C-5), 170.4 (qC, C-1), 159.4 (qC, C-4′), 155.2 (qC, C-4″), 139.7 (qC, C-2), 132.9 (CH, C-2″), 130.4 (CH, C-2′, C-6′), 129.7 (CH, C-6″), 129.6 (qC, C-3), 129.2 (qC, C-3″), 125.2 (qC, C-1″), 123.2 (qC, C-1′), 116.6 (CH, C-3′, C-5′), 115.2 (CH, C-5″), 86.9 (qC, C-4), 71.5 (qC, C-9″), 53.9 (CH_3, 5-OCH_3), 44.7 (CH_2, C-8″), 39.5 (CH_2, C-6), 29.3 (CH_3, C-11″), 29.0 (CH_3, C-10″), 25.8 (CH_2, C-7″).

Butyrolactone VI (**12**): white solid; $[\alpha]_D^{25}$ = +38.2 (*c* 0.1, MeOH); ^{1}H NMR (700 MHz, CD_3OD): δ_H 7.58 (2H, d, J = 8.9 Hz, H-2′, H-6′), 6.87 (2H, d, J = 8.9 Hz, H-3′, H-5′), 6.61 (1H, d, J = 2.0 Hz, H-2″), 6.52 (1H, d, J = 8.2 Hz, H-5″), 6.49 (1H, dd, J = 8.2, 2.0 Hz, H-6″), 3.79 (3H, s, 5-OCH_3), 3.45 (2H, s, H-6), 3.44 (1H, m, H-8″), 2.68

(1H, dd, $J = 14.1, 2.2$ Hz, H_a-7″), 2.54 (1H, dd, $J = 14.1, 10.0$ Hz, H_b-7″), 1.18 (3H, s, H-10″), 1.17 (3H, s, H-11″); ^{13}C NMR (175 MHz, CD_3OD) δ_C 171.6 (qC, C-5), 170.4 (qC, C-1), 159.4 (qC, C-4′), 155.9 (qC, C-4″), 139.8 (qC, C-2), 134.3(CH, C-2″), 130.5 (CH C-6″), 130.4 (CH, C-2′, C-6′), 129.3 (qC, C-3), 127.3 (qC, C-3″), 125.5 (qC, C-1″), 123.1 (qC, C-1′), 116.6 (CH, C-3′, C-5′), 115.9 (CH, C-5″), 86.8 (qC, C-4), 80.6 (CH, C-8″), 73.8 (qC, C-9″), 53.9 (CH_3, 5-OCH_3), 39.5 (CH_2, C-6), 34.0 (CH_2, C-7″), 25.5 (CH_3, C-10″), 25.1 (CH_3, C-11″).

Butyrolactone IV (**13**): yellow oil; $[\alpha]_D^{20}$ = +40.0 (*c* 0.1, MeOH); ^{1}H NMR (700 MHz, CD_3OD) δ_H 7.58 (2H, d, $J = 8.9$ Hz, H-2′, H-6′), 6.87 (2H, d, $J = 8.9$ Hz, H-3′, H-5′), 6.61 (1H, br s, H-2″), 6.54 (1H, dd, $J = 8.2, 1.6$ Hz, H-6″), 6.46 (1H, d, $J = 8.2$ Hz, H-5″), 4.50 (1H, dd, $J = 9.4, 8.5$ Hz, H-8″), 3.79 (3H, s, 5-OCH_3), 3.47 (2H, s, H-6), 3.03 (1H, dd, $J = 15.8, 8.5$ Hz, H_a-7″), 2.97 (1H, dd, $J = 15.8, 9.4$ Hz, H_b-7″), 1.19 (3H, s, H-10″), 1.16 (3H, s, H-11″); ^{13}C NMR (175 MHz, CD_3OD) δ_C 171.5 (qC, C-5), 170.2 (qC, C-1), 160.5 (qC, C-4″), 159.4 (qC, C-4′), 139.7 (qC, C-2), 131.0 (CH, C-6″), 130.4 (CH, C-2′, C-6′), 129.1 (qC, C-3), 128.1 (qC, C-3″), 127.9 (CH C-2″), 126.2 (qC, C-1″), 123.1 (qC, C-1′), 116.6 (CH, C-3′, C-5′), 109.1 (CH, C-5″), 90.4 (CH, C-8″), 86.8 (qC, C-4), 72.5 (qC, C-9″), 53.9 (CH_3, 5-OCH_3), 39.7 (CH_2, C-6), 31.3 (CH_2, C-7″), 25.3 (CH_3, C-10″), 25.2 (CH_3, C-11″).

Table S1. X-ray crystallographic data for **1**

Empirical formula	$C_{16}H_{12}O_4$
Formula weight	269.08
Temperature/K	293(2)
Crystal system	monoclinic
Space group	P2/c
a/Å	23.6601(13)
b/Å	5.4446(3)
c/Å	21.0558(17)
α/°	90
β/°	99.555(7)
γ/°	90
Volume/$Å^3$	2674.8(3)
Z	4
$\rho_{calc}g/cm^3$	1.412
μ/mm^{-1}	0.856
F(000)	1192.0
Crystal size/mm^3	0.4 × 0.1 × 0.05
Radiation	CuKα (λ = 1.54184)
2Θ range for data collection/°	8.518 to 148.942
Index ranges	$-29 \leq h \leq 28$, $-6 \leq k \leq 6$, $-7 \leq l \leq 26$
Reflections collected	5257
Independent reflections	5257 [R_{int} = ?, R_{sigma} = 0.0622]
Data/restraints/parameters	5257/0/389
Goodness-of-fit on F^2	1.061
Final R indexes [I>=2σ (I)]	R_1 = 0.1083, wR_2 = 0.2959
Final R indexes [all data]	R_1 = 0.1280, wR_2 = 0.3097
Largest diff. peak/hole / e $Å^{-3}$	0.62/-0.48

Table S2. The primer sequences for the genes.

Gene	Forward(5'-3' sequence)	Reverse (5'-3' sequence)
GAPDH	GCTCTCTGCTCCTCCTGTTC	ACGACCAAATCCGTTGACTC
LXRα	TCAGCATCTTCTCTGCAGACCGG	TCATTAGCATCCGTGGGAACA
ABCA1	ACCCACCCTATGAACAACATGA	GAGTCGGGTAACGGAAACAGG
UGT1A1	CATGCTGGGAAGATACTGTTGAT	GCCCGAGACTAACAAAAGACTCT
TNFα	CAAGCCTGTAGCCCATGTTGTA	ACCAGCTGGTTATCTCTCAGCT
CXCL10	GTGGCATTCAAGGAGTACCTC	TGATGGCCTTCGATTCTGGATT
CYP3A5	AATGTTTTGTCCTATCGTCAGGG	AGACCTTCGATTTGTGAAGACAG
CYP3A7	AAACTTGGCCGTGGAAACCT	CAGCATAGGCTGTTGACAGTC
ALDH3A1	CTCTGTGACCCCTCGATCCA	GCATCTTCCCCGTAGAACTCTT
UGT2B10	GAAATGGACTACAGTTCTGCTGA	GTGGATGAGTCGTTGGGATCA
CYP1A1	TCGGCCACGGAGTTTCTTC	GGTCAGCATGTGCCCAATCA
GSTA2	TACTCCAATATACGGGGCAGAA	TCCTCAGGTTGACTAAAGGGC
gapdh	CTTTGGCATTGTGGAAGGGC	TGCAGGGATGATGTTCTGGG
Il6	TAGTCCTTCCTACCCCAATTTCC	TTGGTCCTTAGCCACTCCTTC
Cxcl10	CTCATCCTGCTGGGTCTGAG	CCTATGGCCCTCATTCTCAC
Bcl2	GCTACCGTCGTGACTTCGC	CCCCACCGAACTCAAAGAAGG
Bax	AGACAGGGGCCTTTTTGCTAC	AATTCGCCGGAGACACTCG
Pcna	TTGCACGTATATGCCGAGACC	GGTGAACAGGCTCATTCATCTCT
Tnfα	GACGTGGAACTGGCAGAAGAG	TTGGTGGTTTGTGAGTGTGAG
Ccl2	CCTGCTGTTCACAGTTGCC	ATTGGGATCATCTTGCTGGT
Lxrα	AGGAGTGTCGACTTCGCAAA	CTCTTCTTGCCGCTTCAGTTT

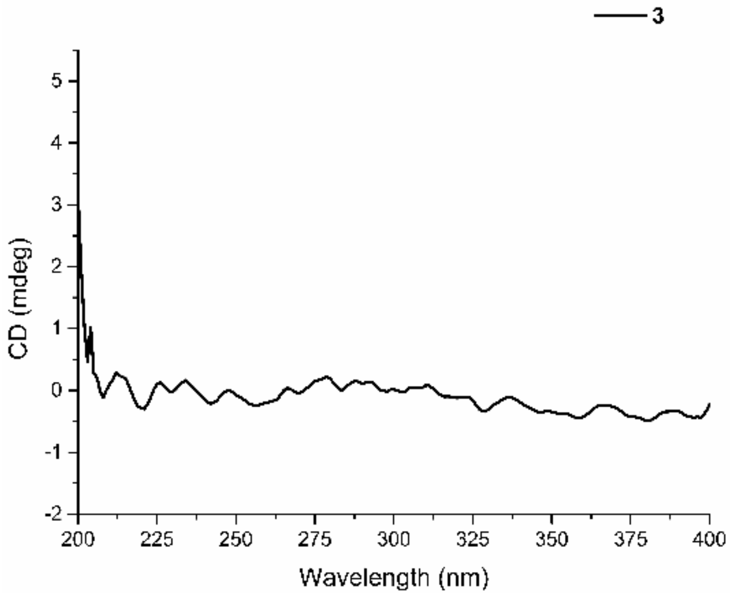

Figure S1. Experimental ECD spectrum of **3**.

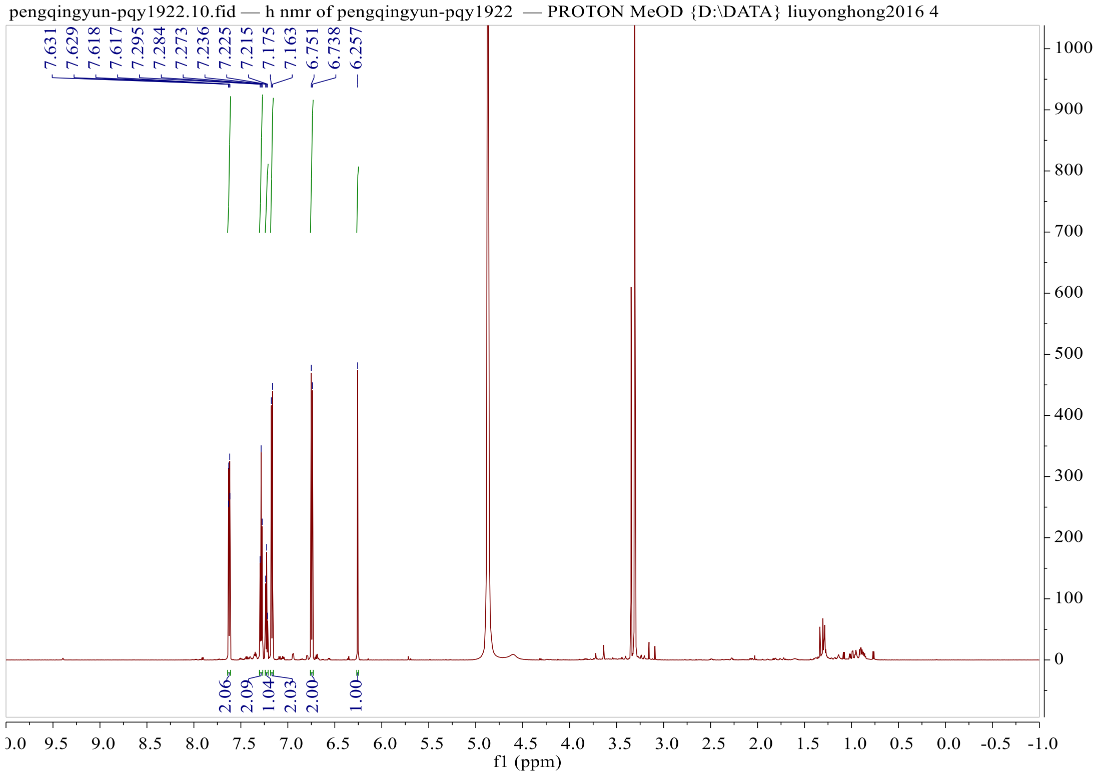

Figure S2. ^{1}H NMR spectrum of **1** (CD_3OD, 700 MHz)

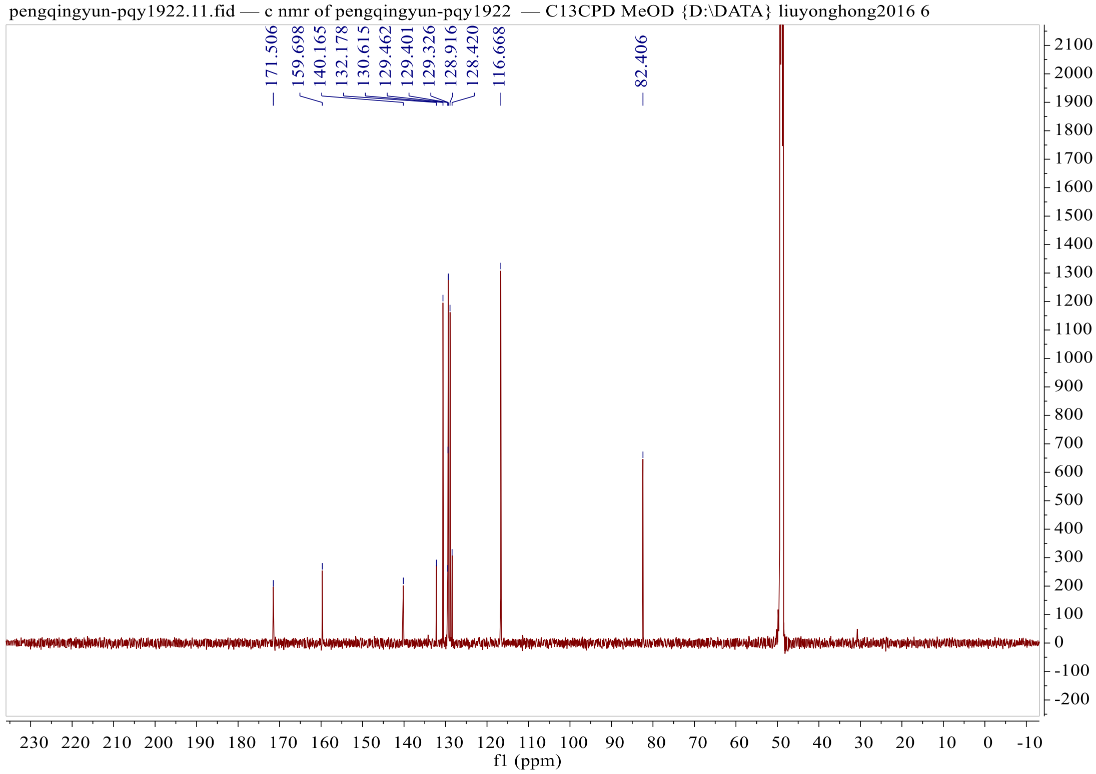

Figure S3. ^{13}C NMR spectrum of **1** (CD_3OD, 175 MHz).

.

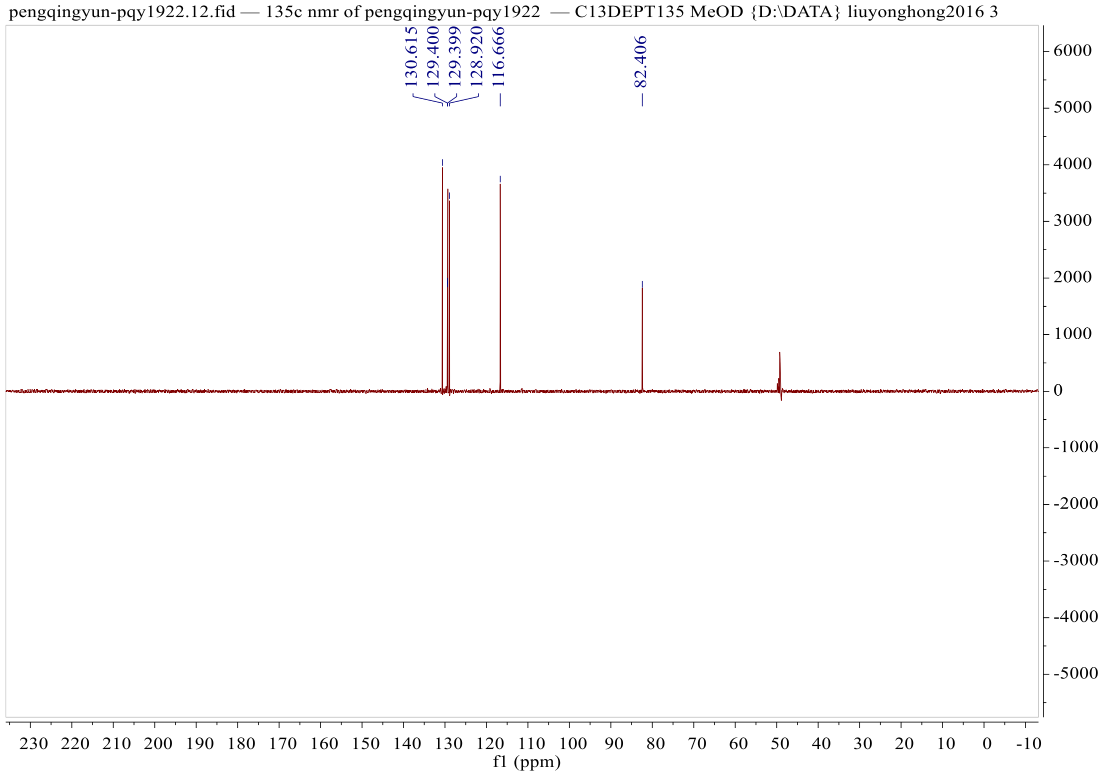

Figure S4. DEPT spectrum of **1** (CD_3OD).

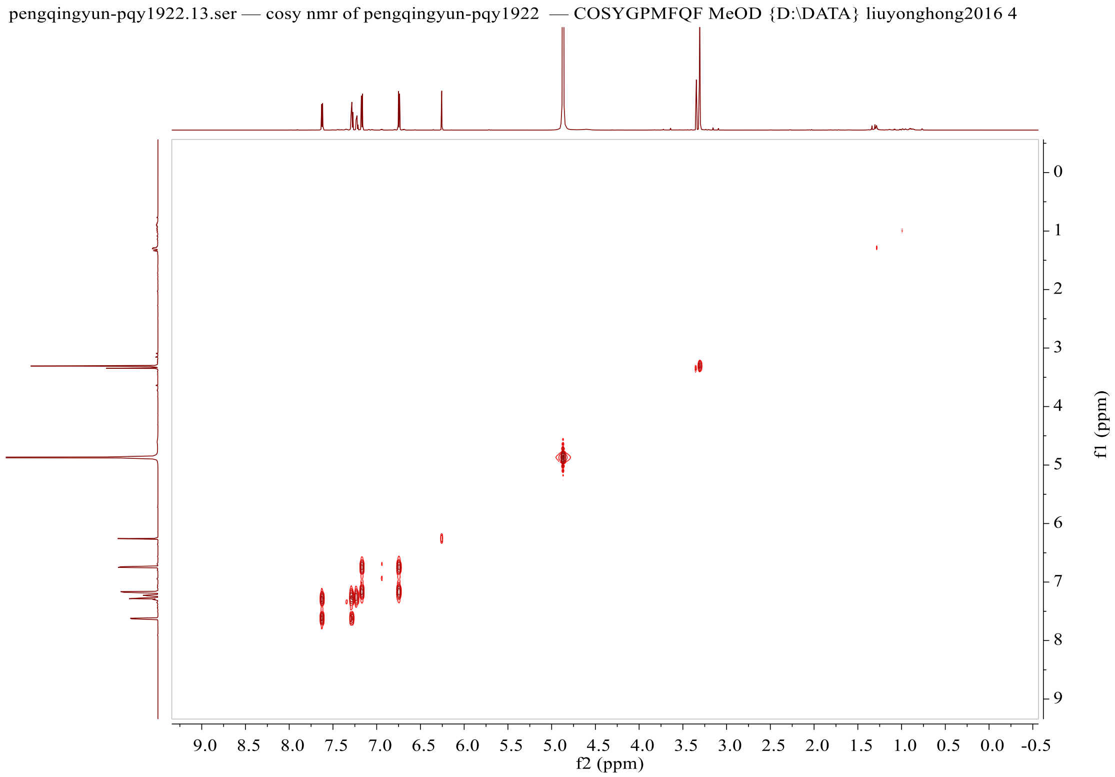

Figure S5. COSY spectrum of **1** (CD_3OD).

pengqingyun-pqy1922.15.ser — qc nmr of pengqingyun-pqy1922 — HSQCEDETGP MeOD {D:\DATA} liuyonghong2016 4

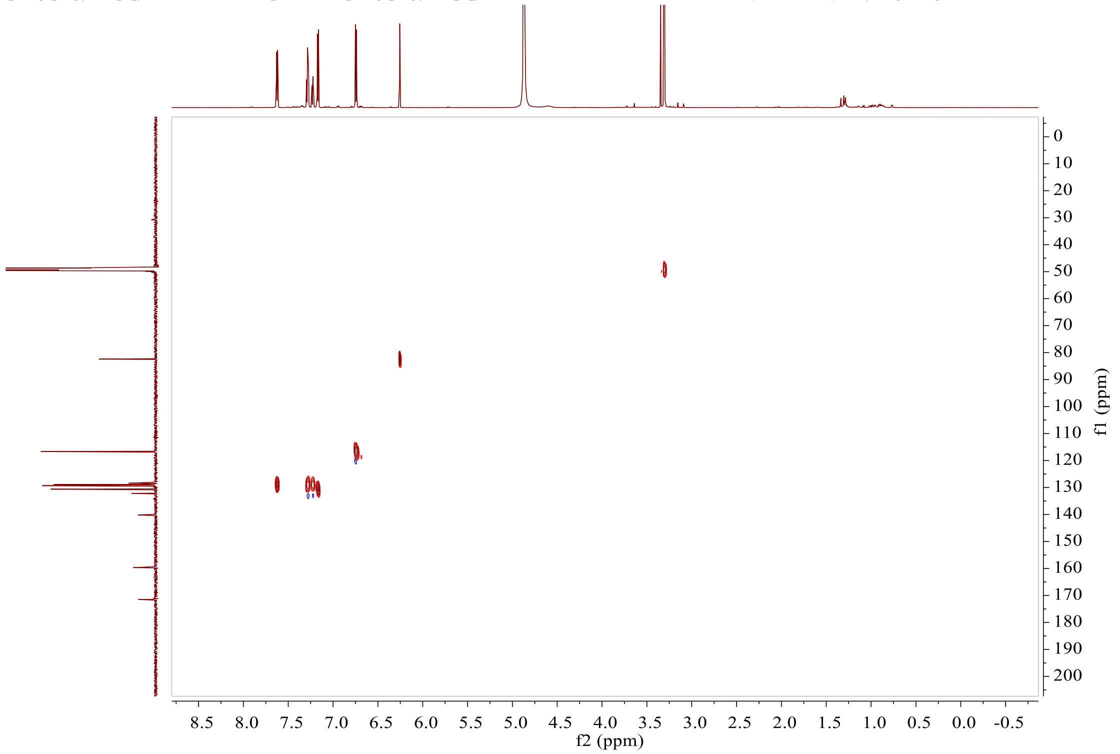

Figure S6. HSQC spectrum of **1** (CD_3OD).

pengqingyun-pqy1922.16.ser — bc nmr of pengqingyun-pqy1922 — hmbcetgpl3nd MeOD {D:\DATA} liuyonghong2016 4

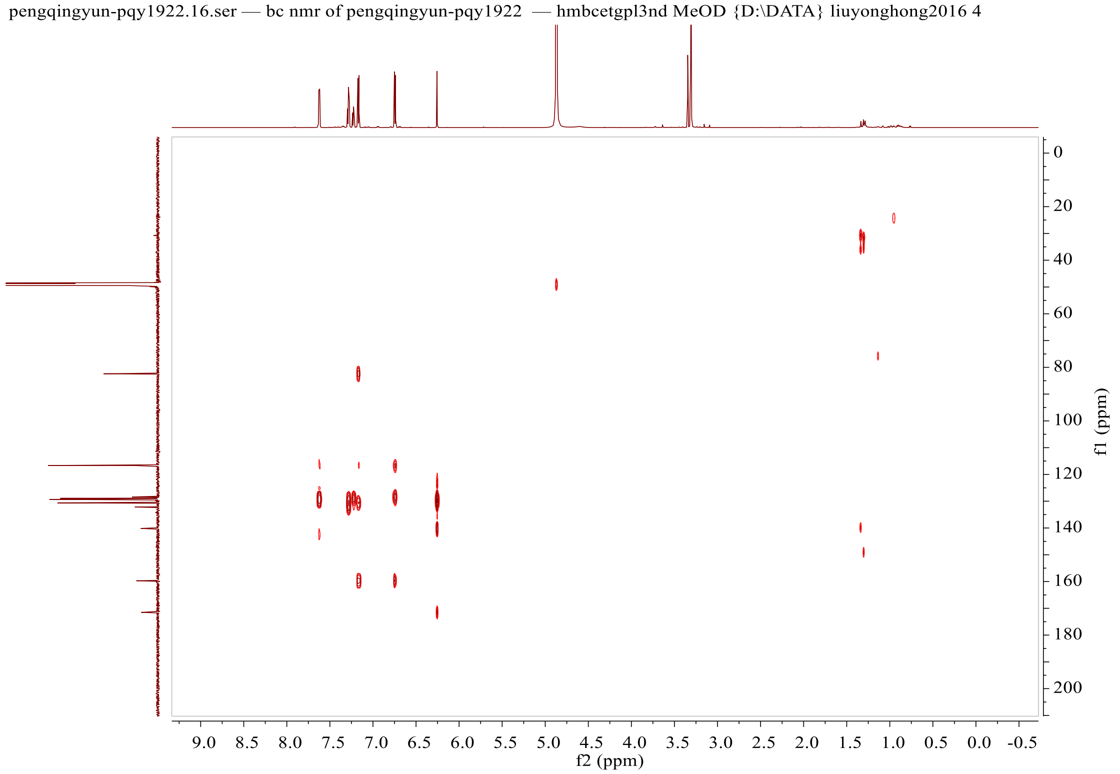

Figure S7. HMBC spectrum of **1** (CD_3OD).

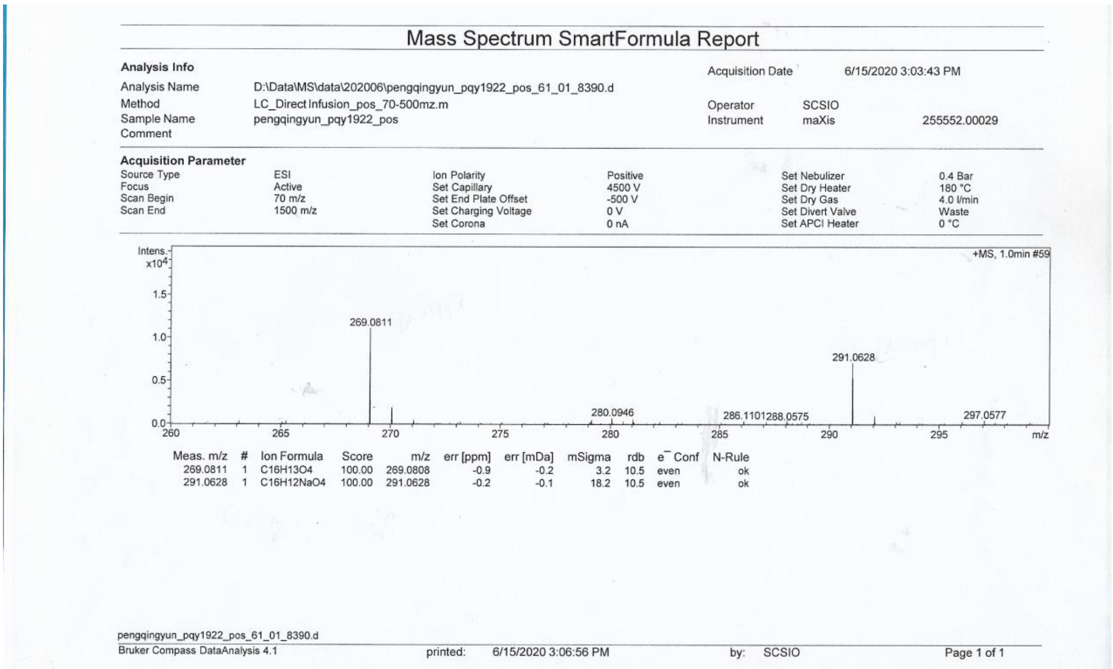

Figure S8. HRESIMS spectrum of **1**.

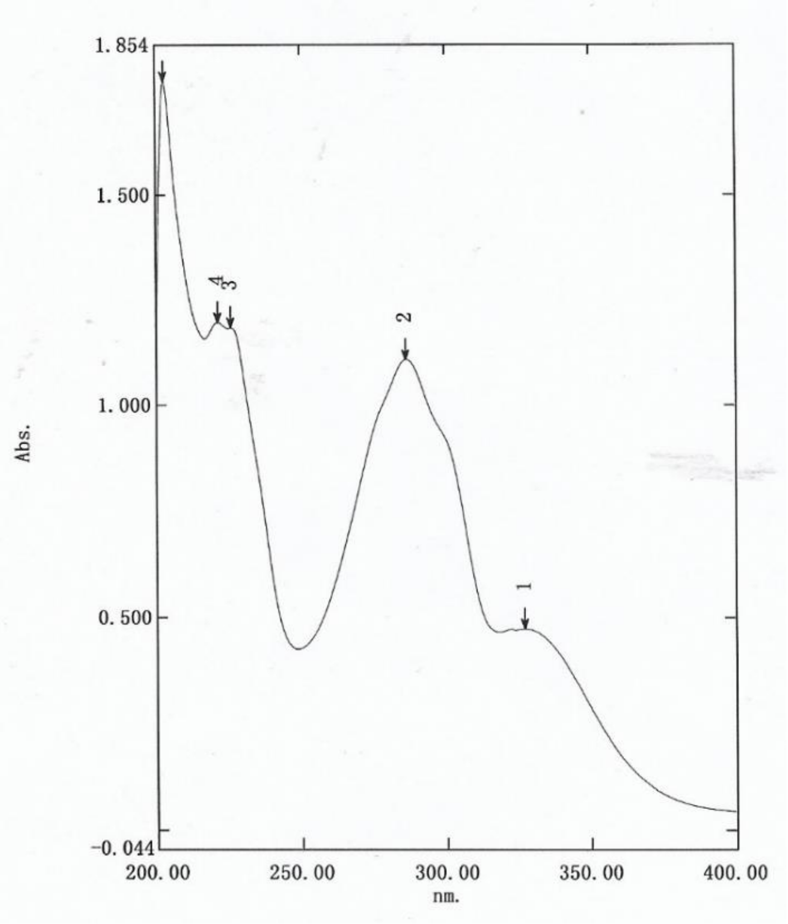

Figure S9. UV spectrum of **1**.

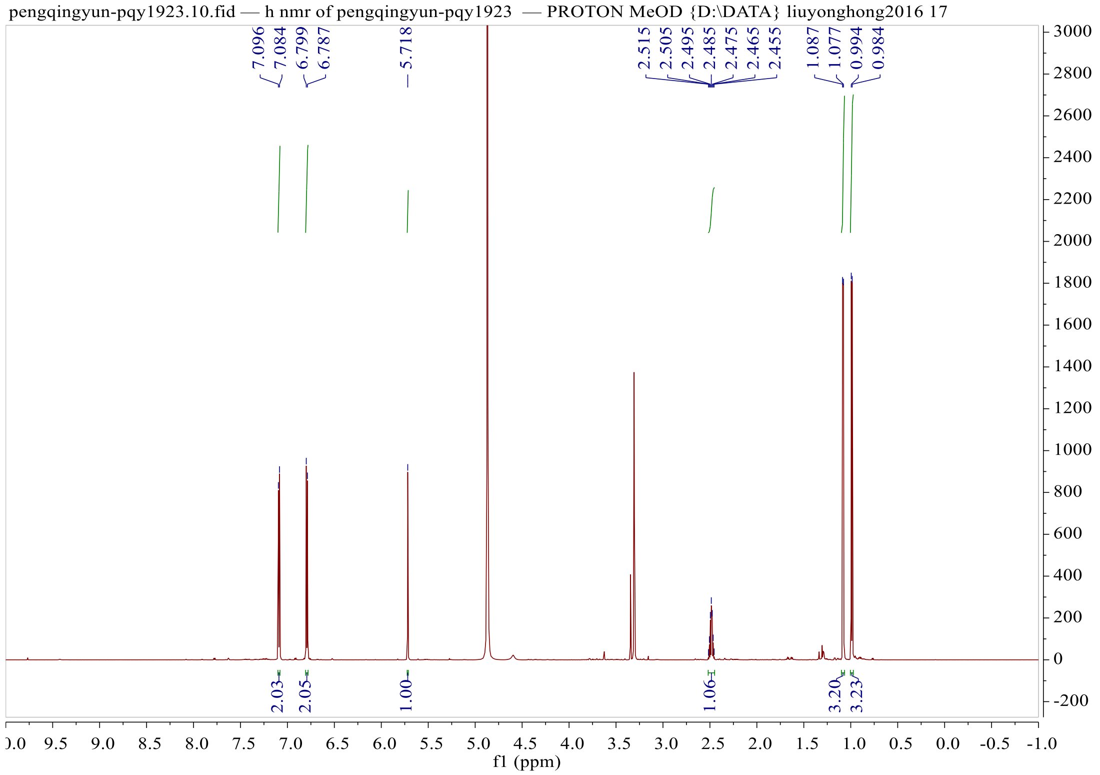

Figure S10. ^{1}H NMR spectrum of **2** (CD_3OD, 700 MHz).

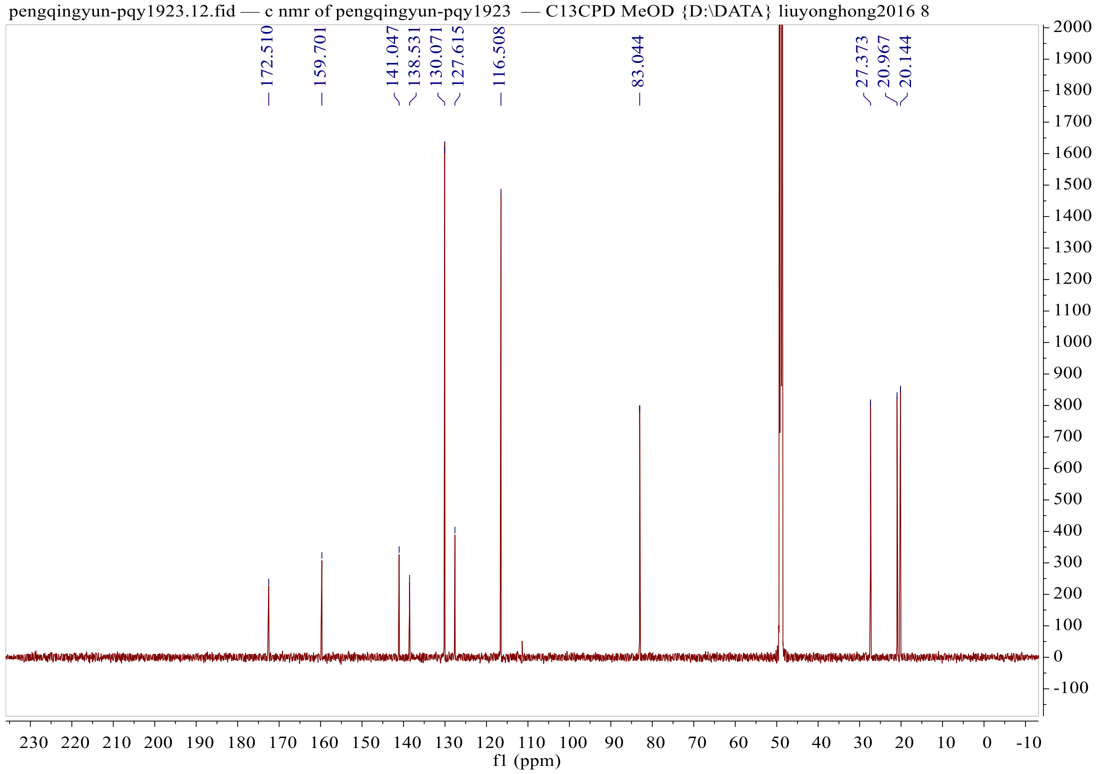

Figure S11. ^{13}C NMR spectrum of **2** (CD_3OD, 175 MHz).

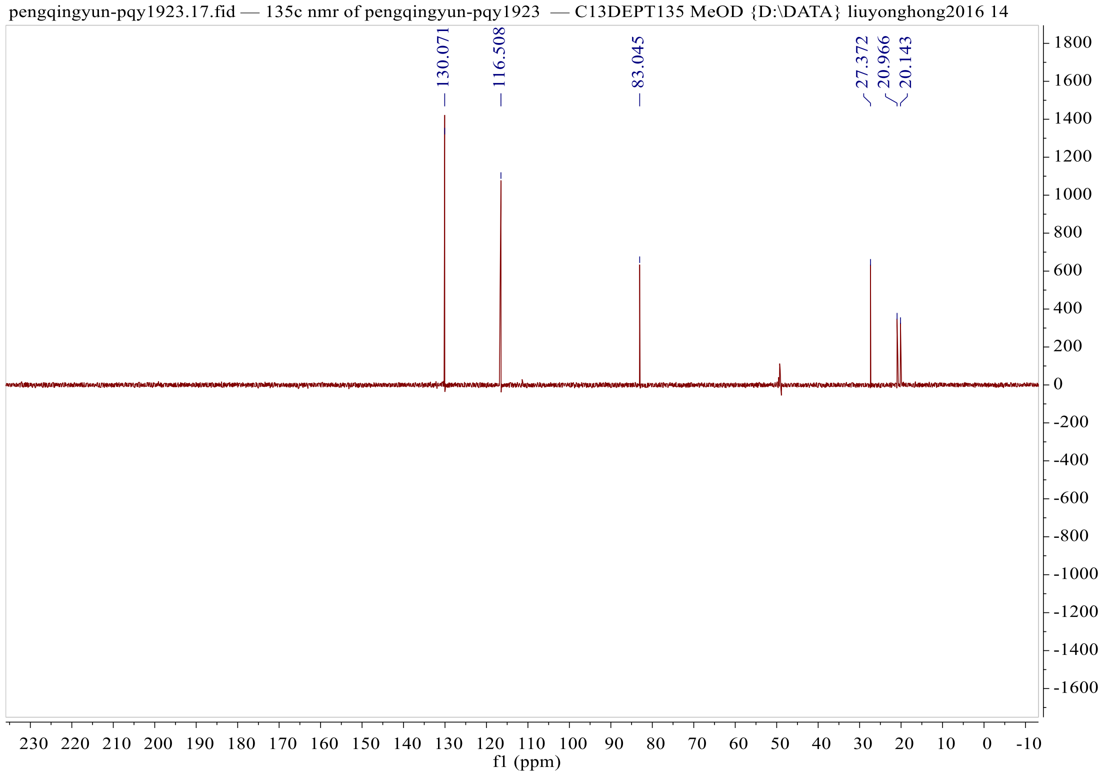

Figure S12. DEPT spectrum of **2** (CD_3OD).

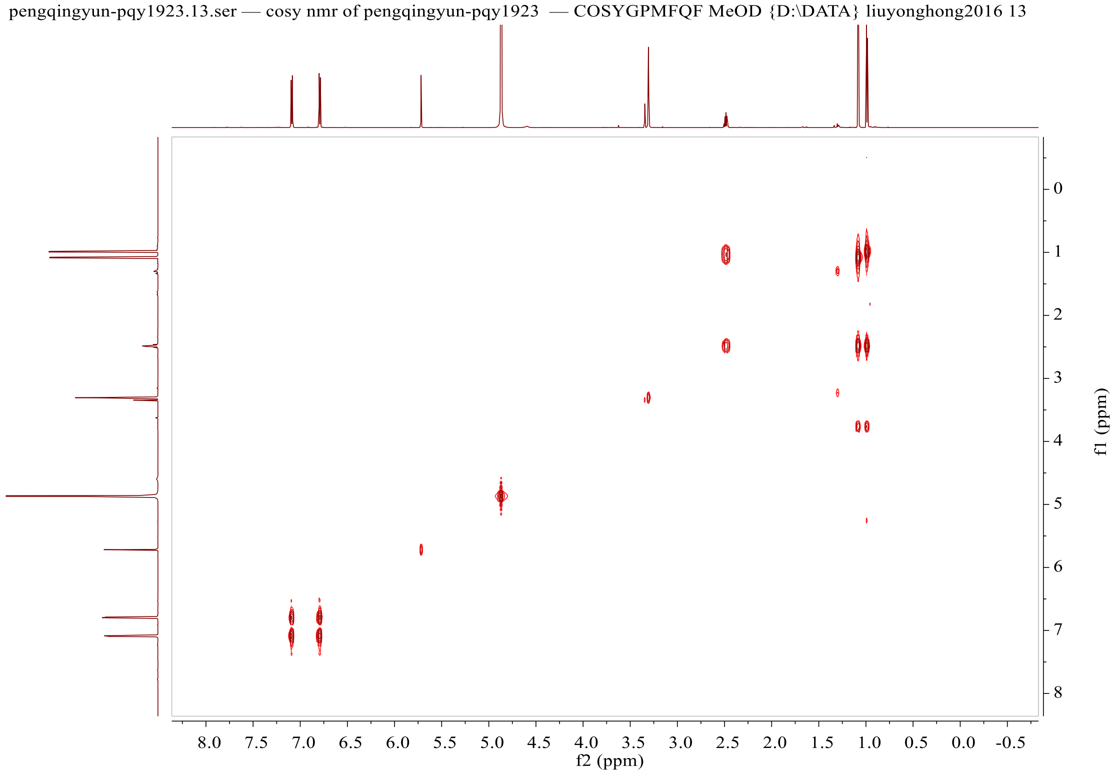

Figure S13. COSY spectrum of **2** (CD_3OD).

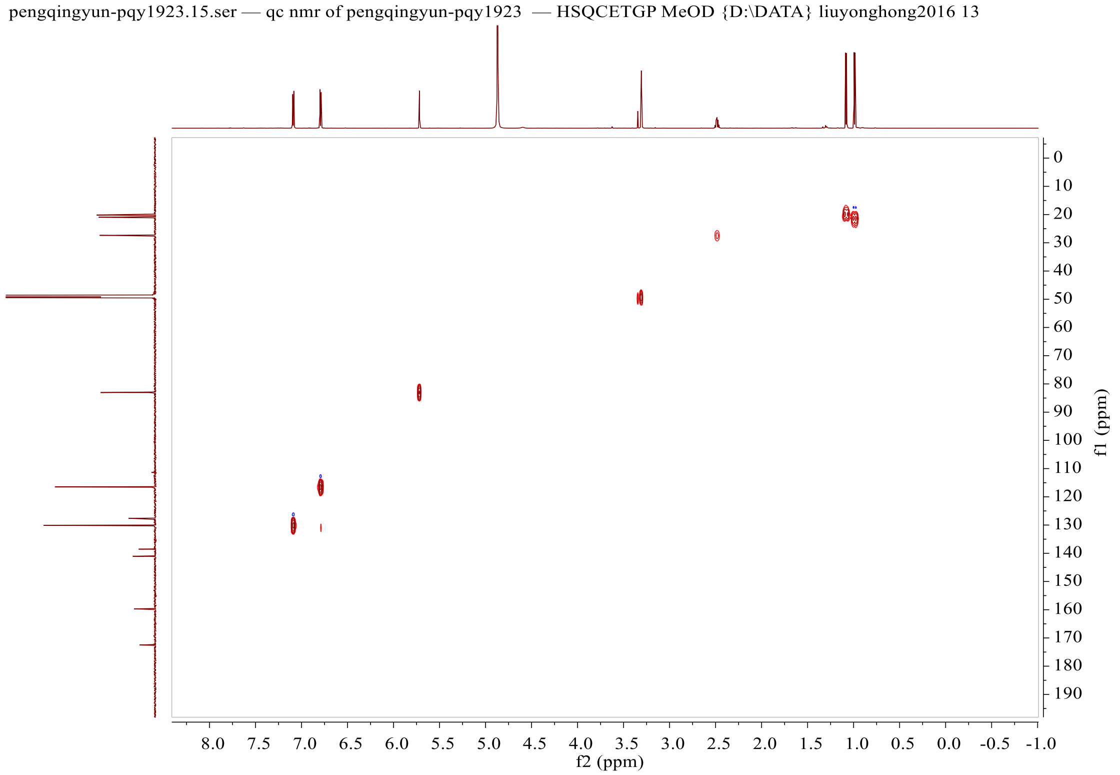

Figure S14. HSQC spectrum of **2** (CD_3OD).

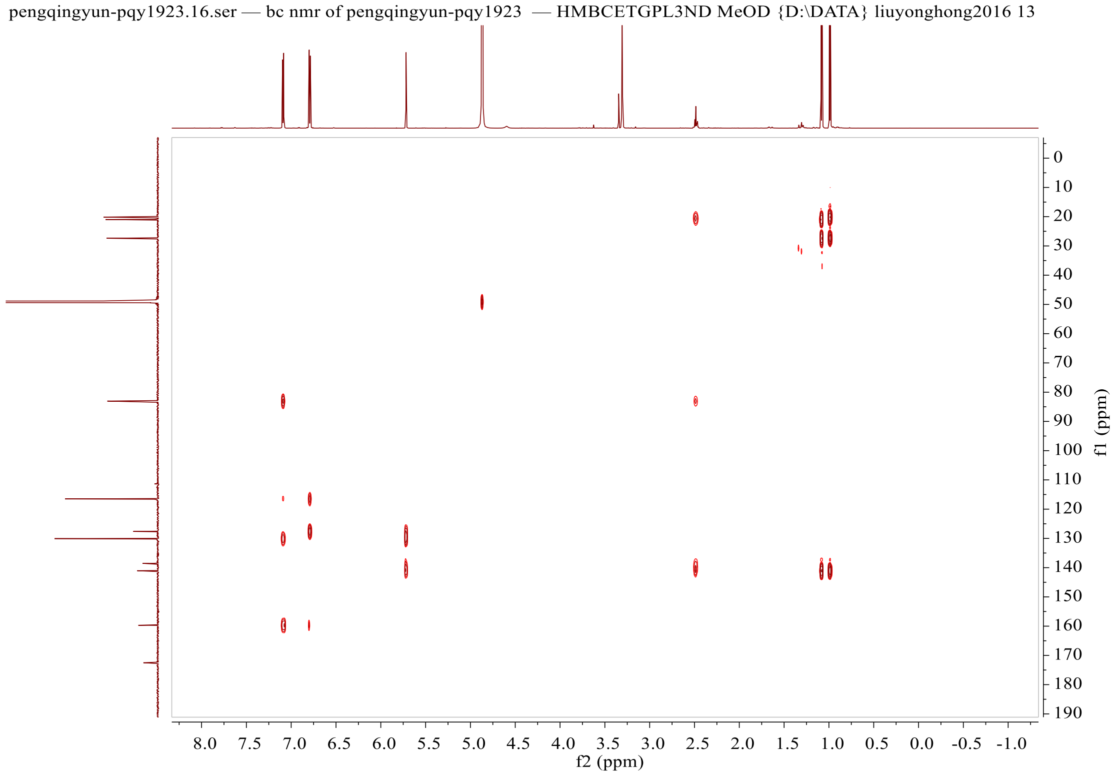

Figure S15. HMBC spectrum of **2** (CD_3OD).

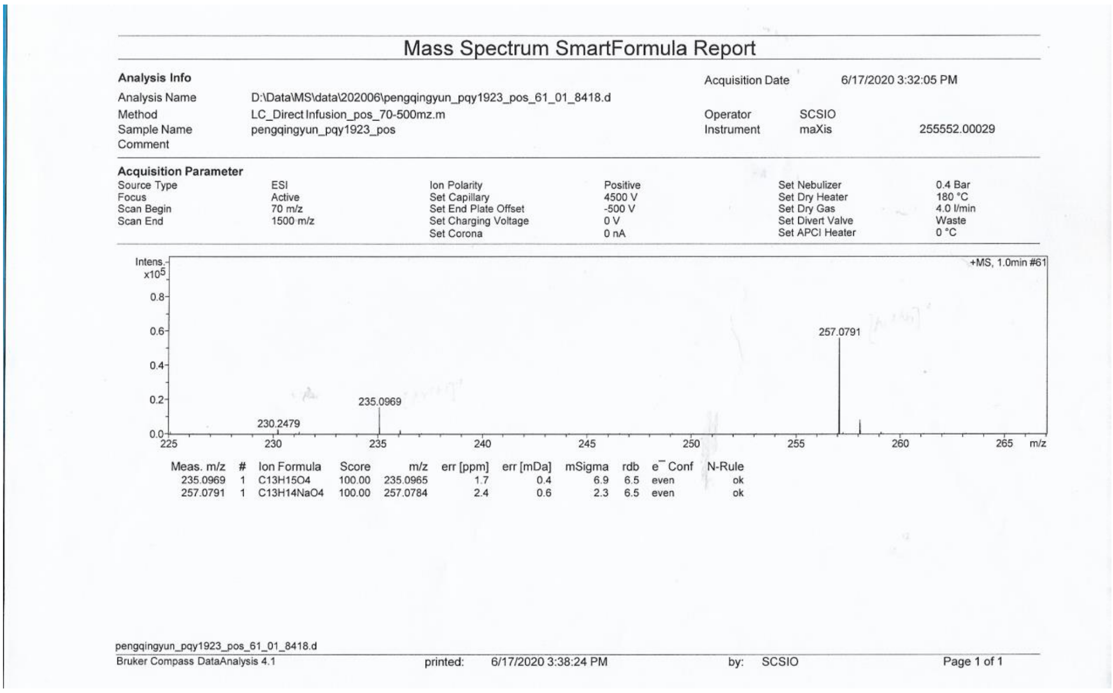

Figure S16. HRESIMS spectrum of **2**.

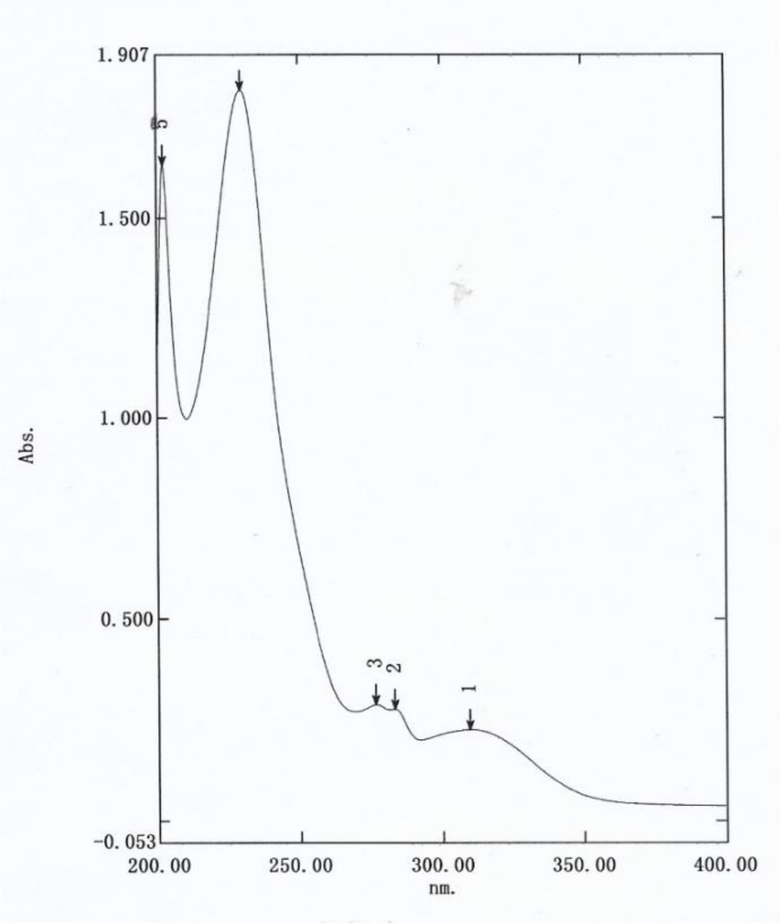

Figure S17. UV spectrum of **2**.

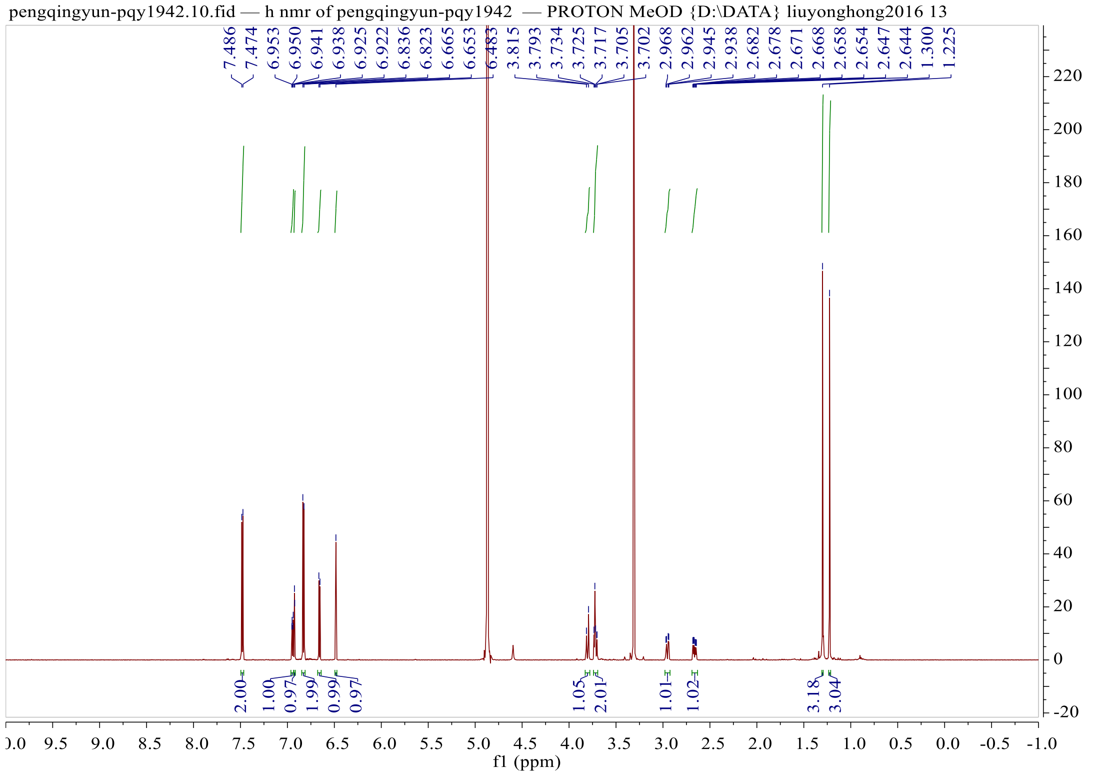

Figure S18. ^{1}H NMR spectrum of **3** (CD_3OD, 700 Hz).

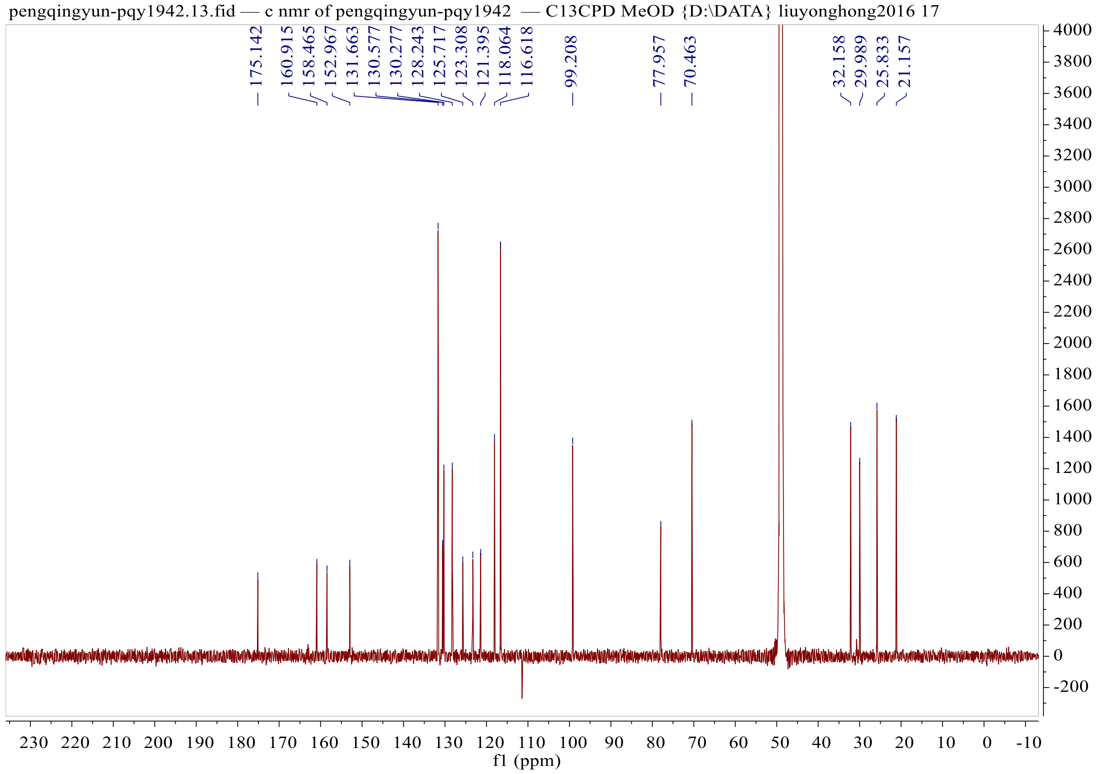

Figure S19. ^{13}C NMR spectrum of **3** (CD_3OD, 175 Hz).

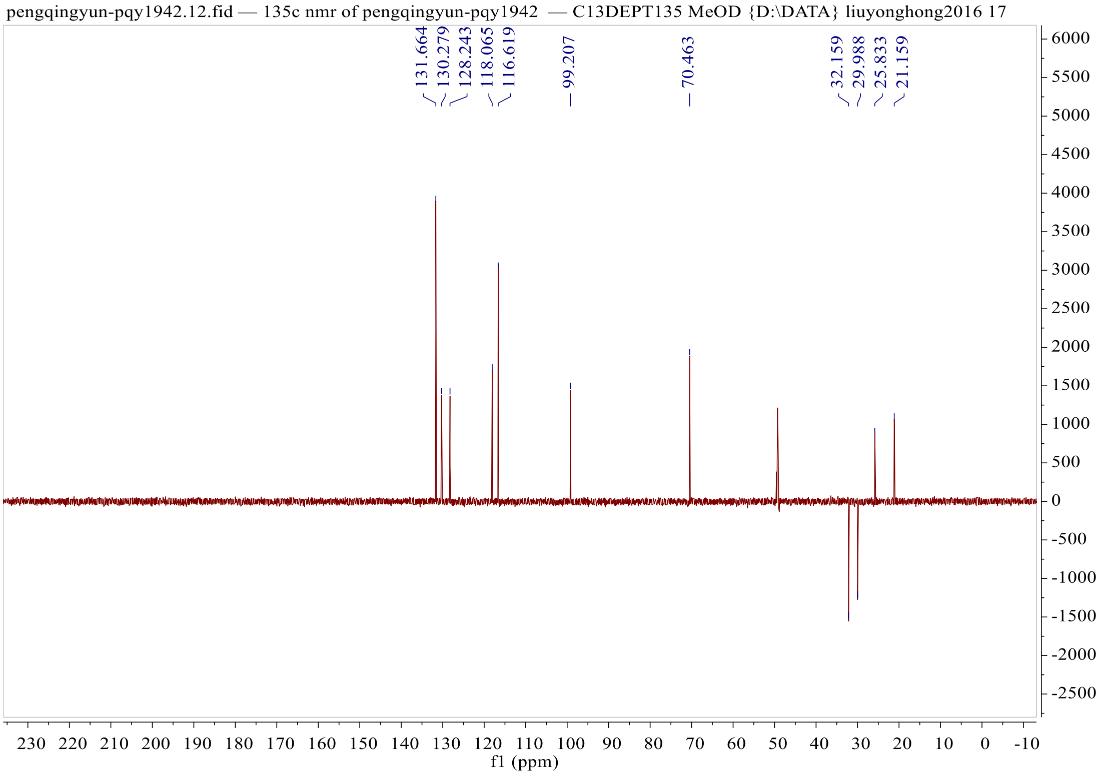

Figure S20. DEPT spectrum of **3** (CD_3OD).

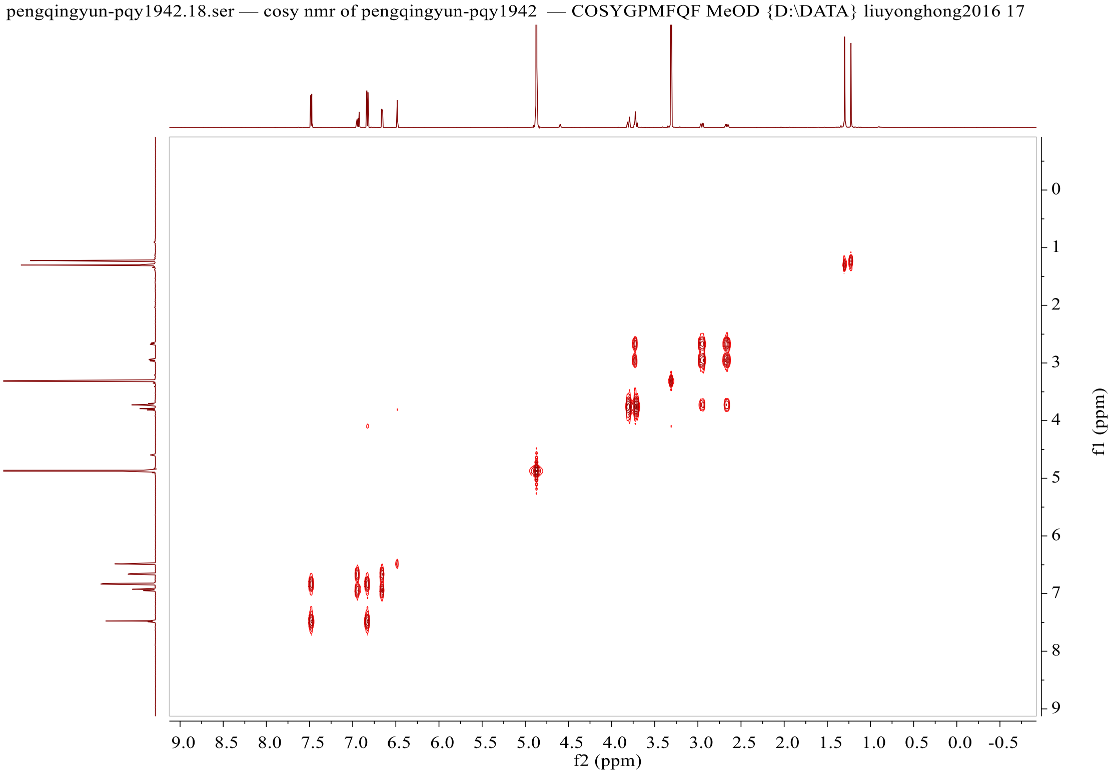

Figure S21. COSY of spectrum of **3** (CD_3OD).

pengqingyun-pqy1942.16.ser — qc nmr of pengqingyun-pqy1942 — HSQCETGP MeOD {D:\DATA} liuyonghong2016 18

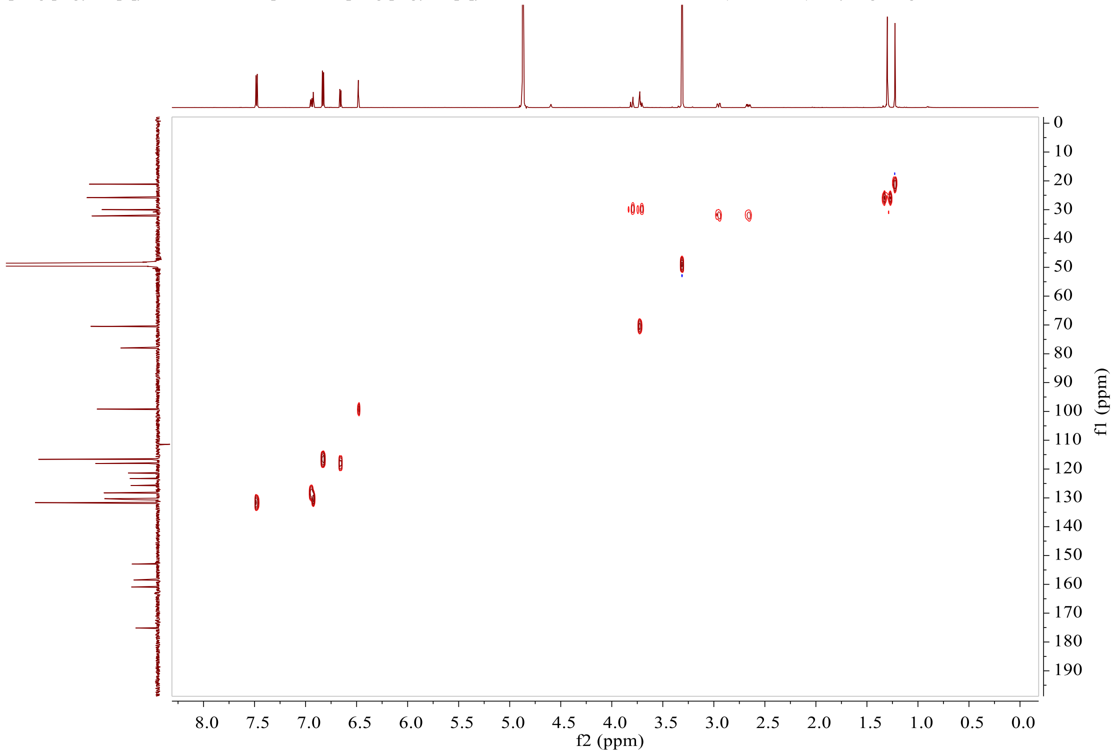

Figure S22. HSQC of spectrum of **3** (CD_3OD).

pengqingyun-pqy1942.15.ser — bc nmr of pengqingyun-pqy1942 — HMBCETGPL3ND MeOD {D:\DATA} liuyonghong2016 18

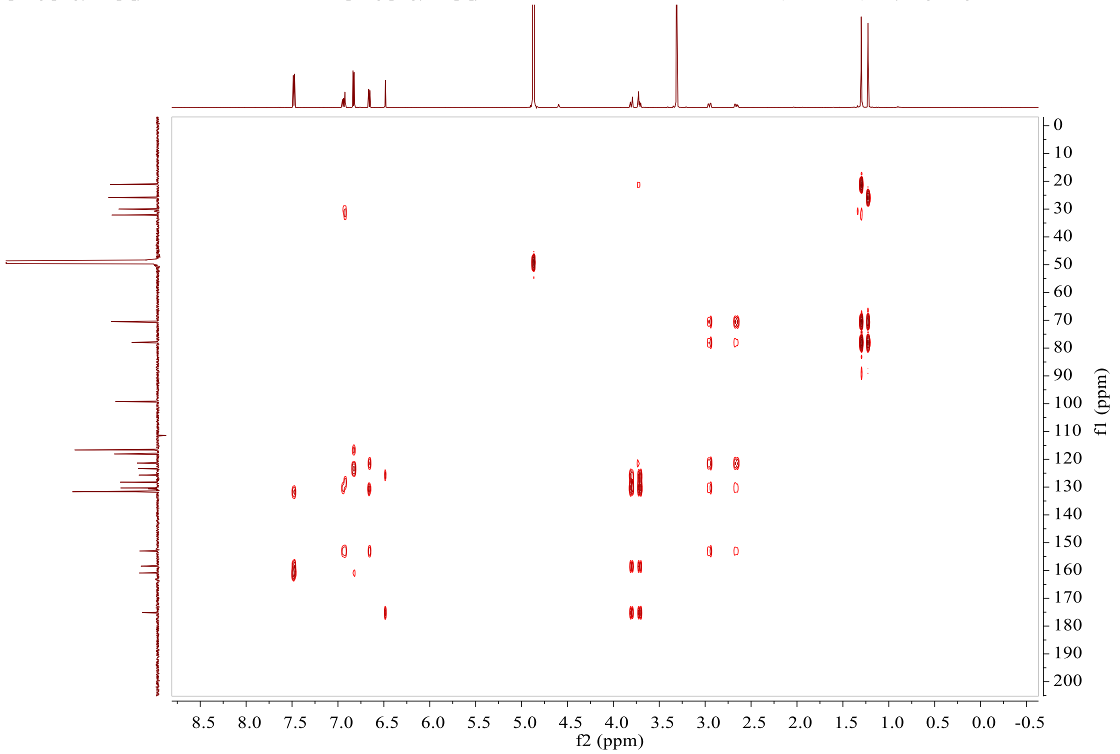

Figure S23. HMBC of spectrum of **3** (CD_3OD).

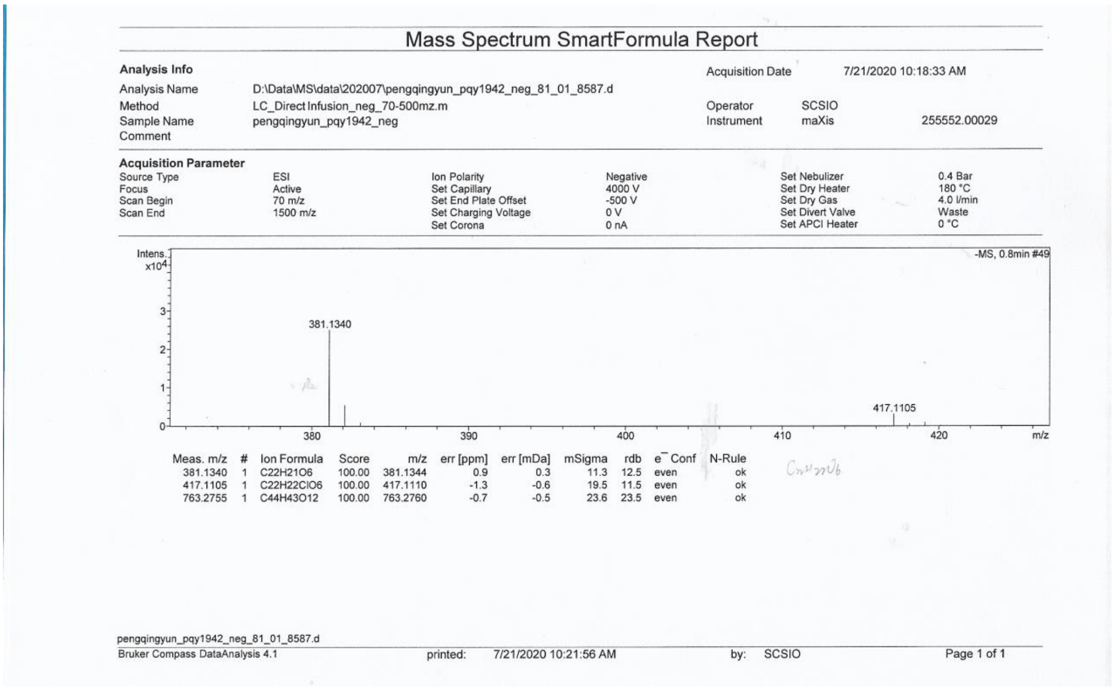

Mass Spectrum SmartFormula Report

Analysis Info		Acquisition Date	7/21/2020 10:18:33 AM	
Analysis Name	D:\Data\MS\data\202007\pengqingyun_pqy1942_neg_81_01_8587.d			
Method	LC_Direct Infusion_neg_70-500mz.m	Operator	SCSIO	
Sample Name	pengqingyun_pqy1942_neg	Instrument	maXis	255552.00029
Comment				

Acquisition Parameter

Source Type	ESI	Ion Polarity	Negative	Set Nebulizer	0.4 Bar
Focus	Active	Set Capillary	4000 V	Set Dry Heater	180 °C
Scan Begin	70 m/z	Set End Plate Offset	-500 V	Set Dry Gas	4.0 l/min
Scan End	1500 m/z	Set Charging Voltage	0 V	Set Divert Valve	Waste
		Set Corona	0 nA	Set APCI Heater	0 °C

Meas. m/z	#	Ion Formula	Score	m/z	err [ppm]	err [mDa]	mSigma	rdb	e⁻ Conf	N-Rule
381.1340	1	C22H21O6	100.00	381.1344	0.9	0.3	11.3	12.5	even	ok
417.1105	1	C22H22ClO6	100.00	417.1110	-1.3	-0.6	19.5	11.5	even	ok
763.2755	1	C44H43O12	100.00	763.2760	-0.7	-0.5	23.6	23.5	even	ok

pengqingyun_pqy1942_neg_81_01_8587.d
Bruker Compass DataAnalysis 4.1 printed: 7/21/2020 10:21:56 AM by: SCSIO Page 1 of 1

Figure S24. HRESIMS spectrum of **3**.

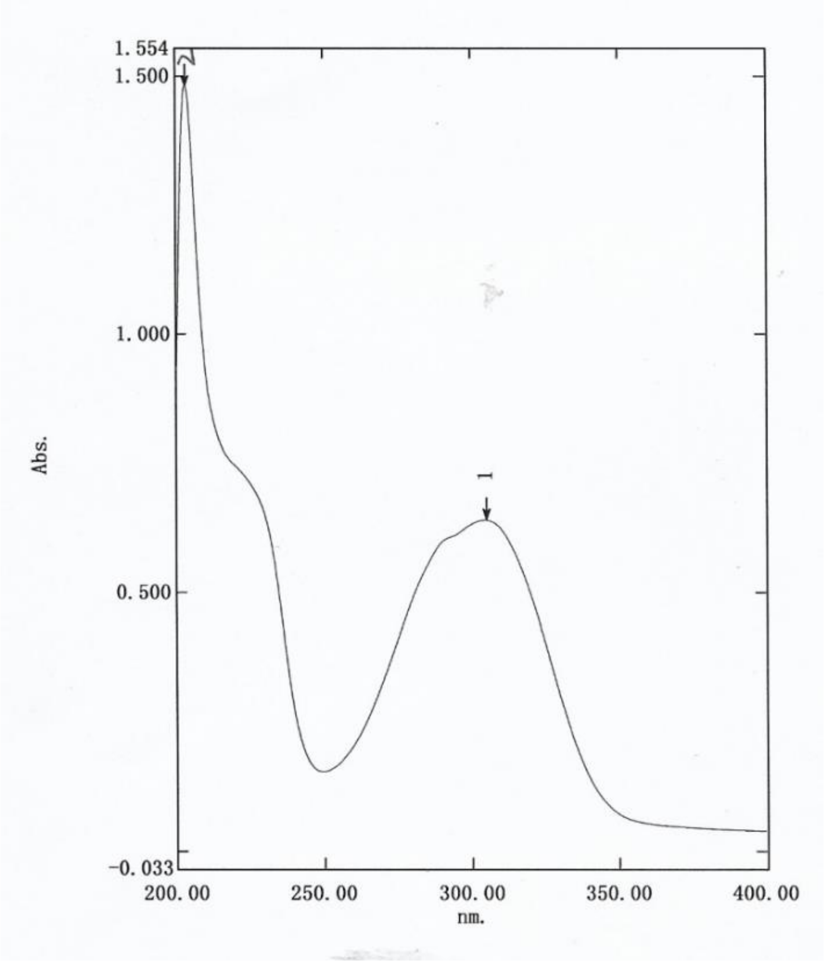

Figure S25. UV spectrum of **3**.

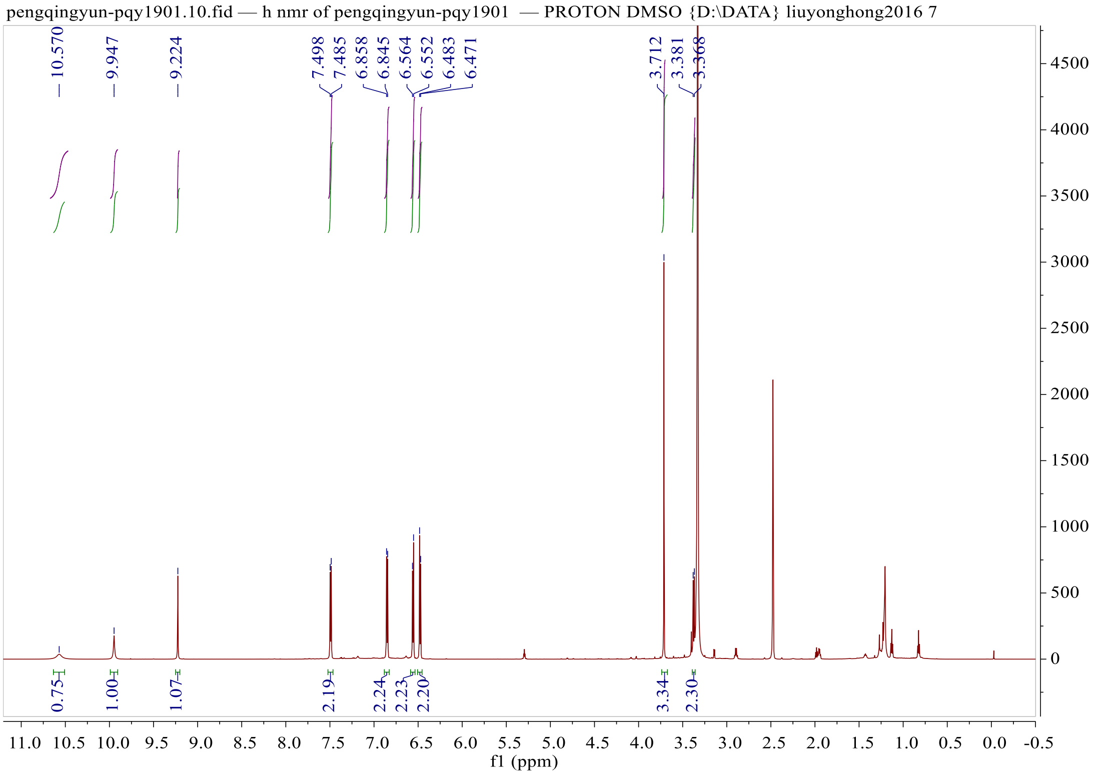

Figure S26. ^{1}H NMR spectrum of **4** (DMSO-d_6, 700 MHz).

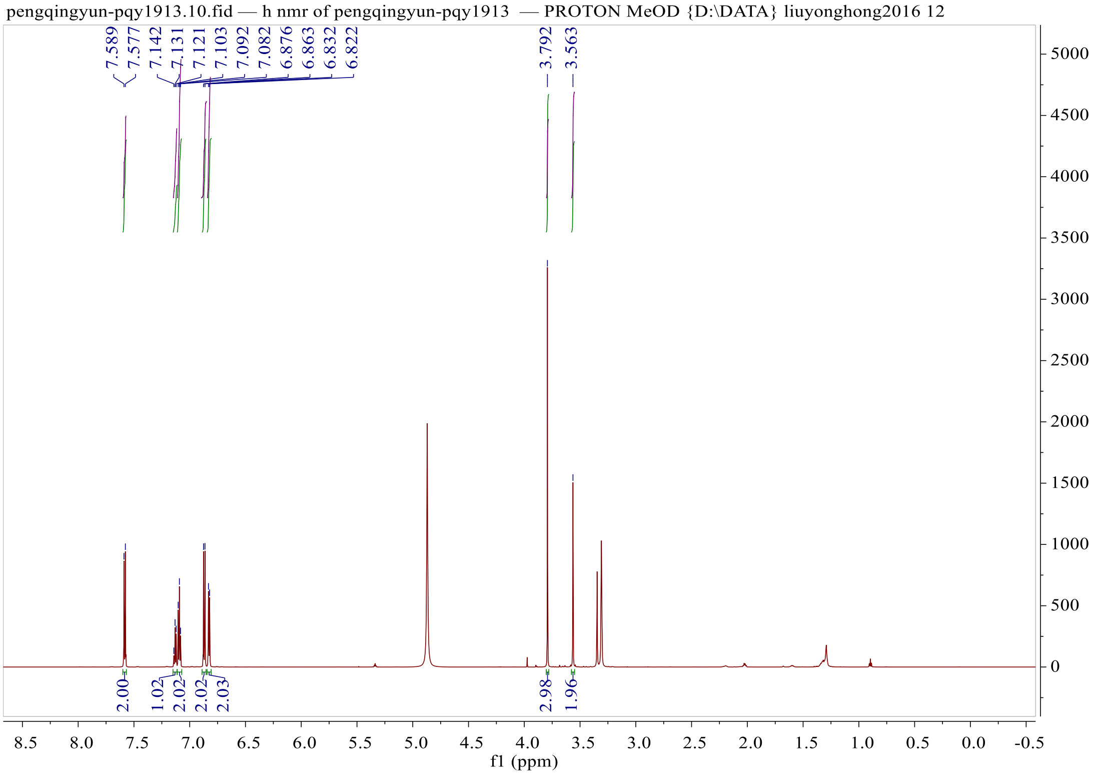

Figure S27. ^{1}H NMR spectrum of **5** (CD_3OD, 700 MHz).

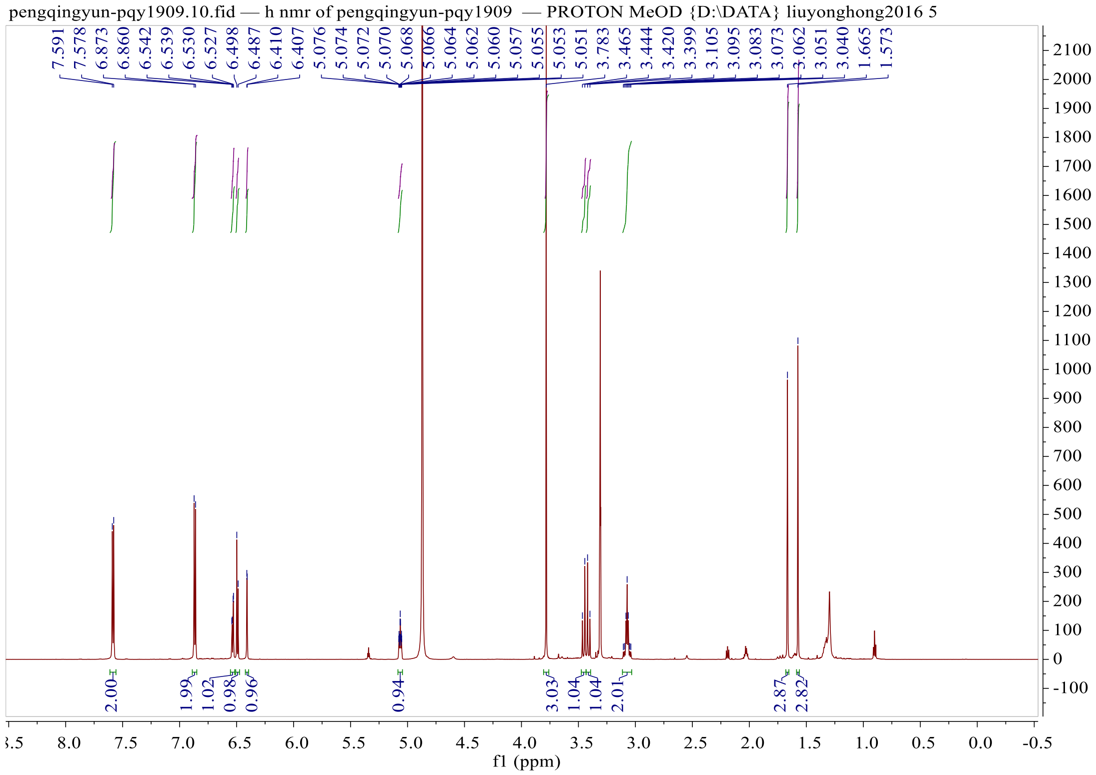

Figure S28. ^{1}H NMR spectrum of **6** (CD_3OD, 700 MHz).

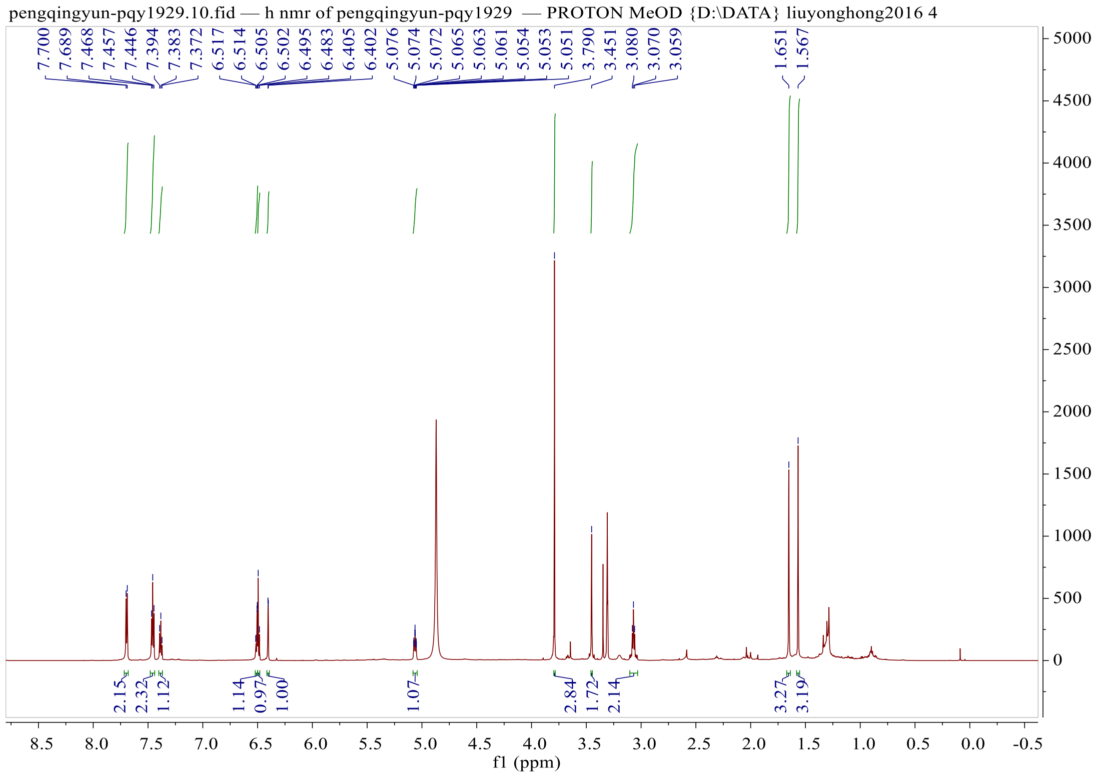

Figure S29. ^{1}H NMR spectrum of **7** (CD_3OD, 700 MHz).

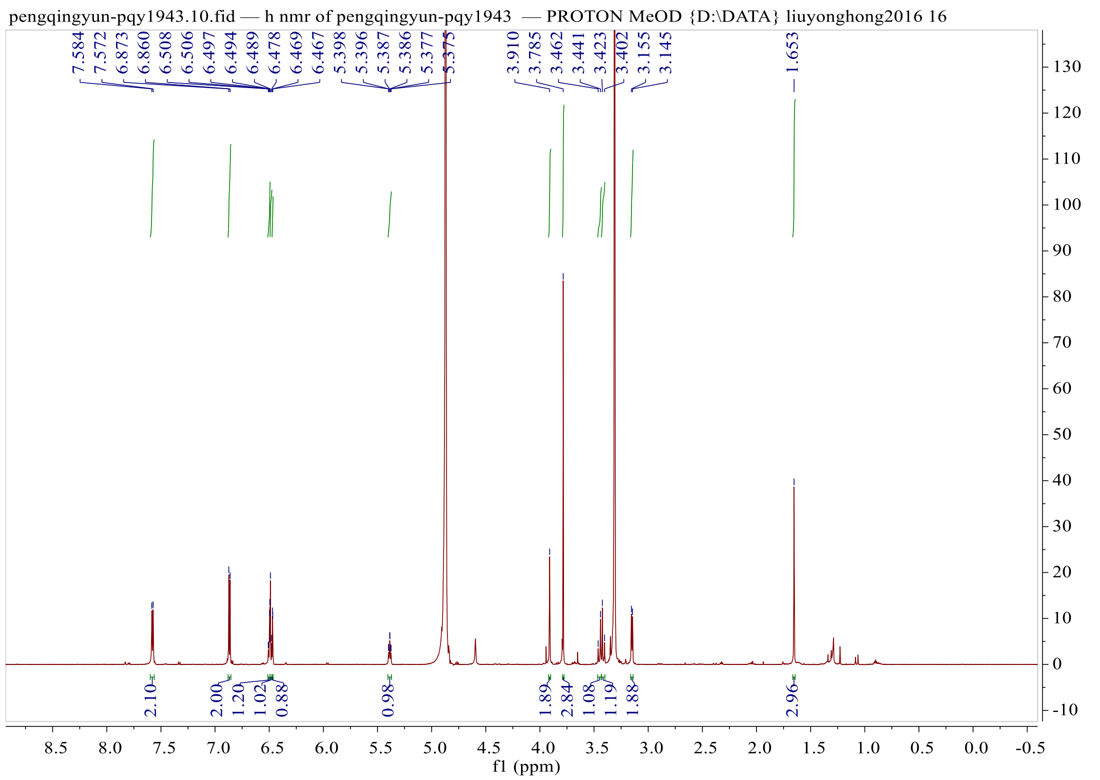

Figure S30. ^{1}H NMR spectrum of **8** (CD_3OD, 700 MHz).

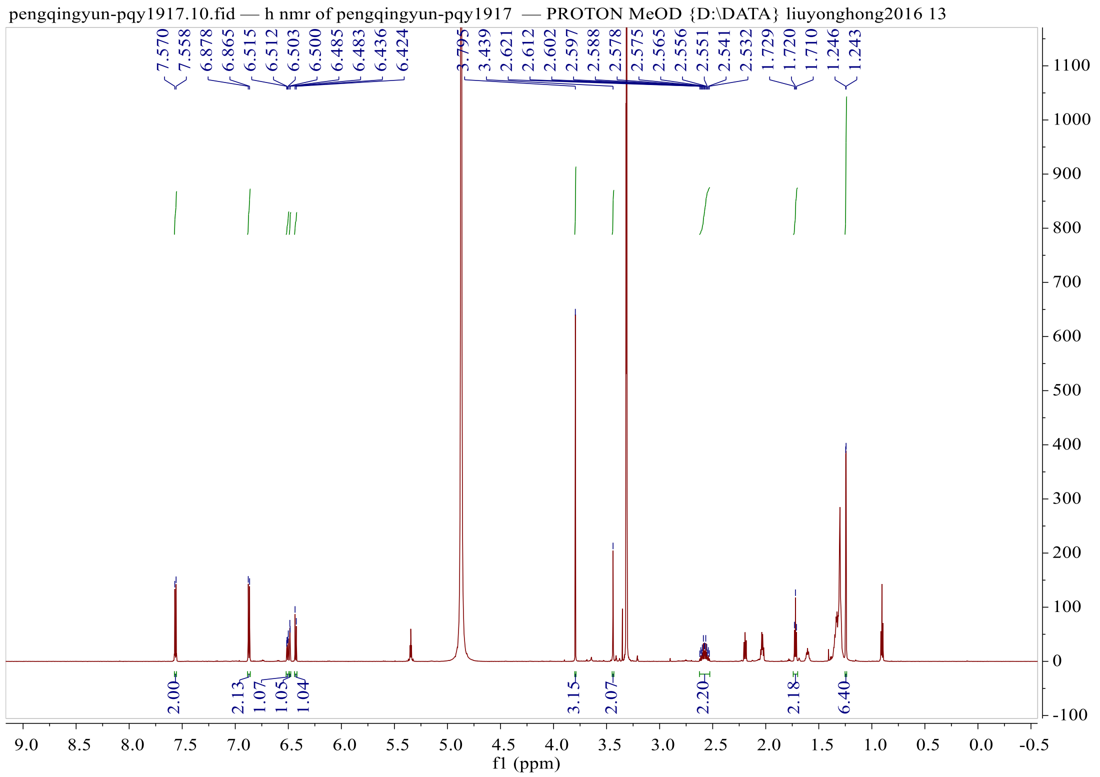

Figure S31. ^{1}H NMR spectrum of **9** (CD_3OD, 700 MHz).

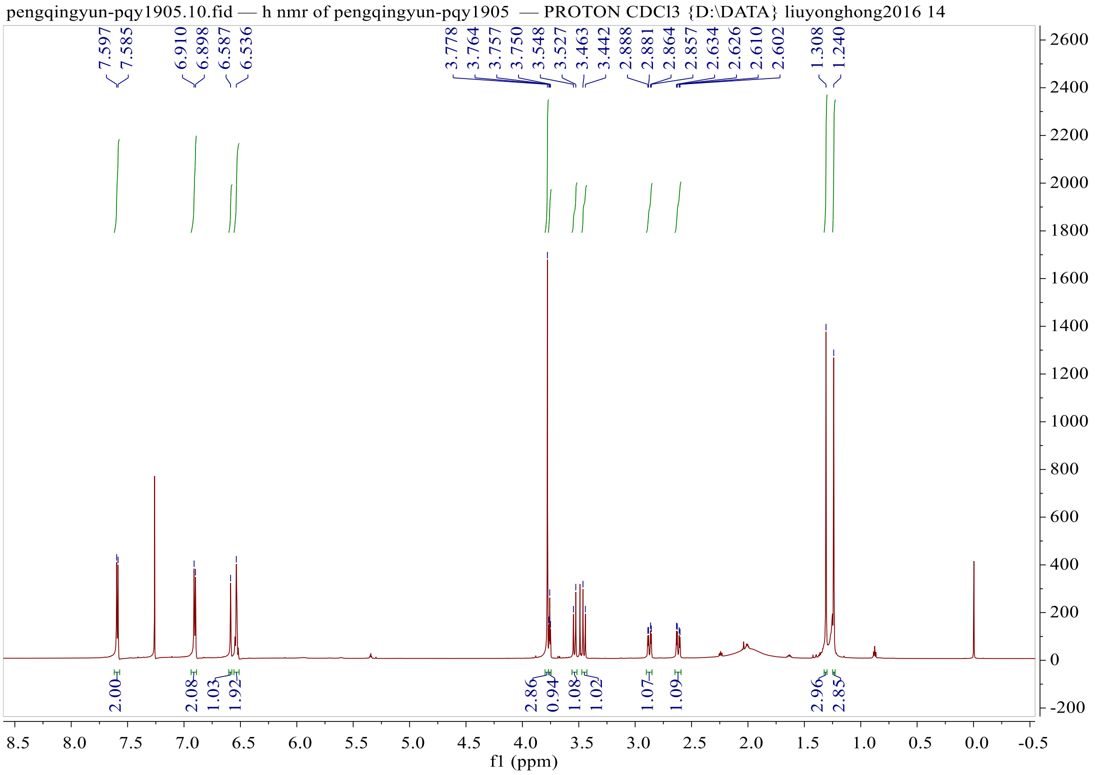

Figure S32. ^{1}H NMR spectrum of **10** ($CDCl_3$, 700 MHz).

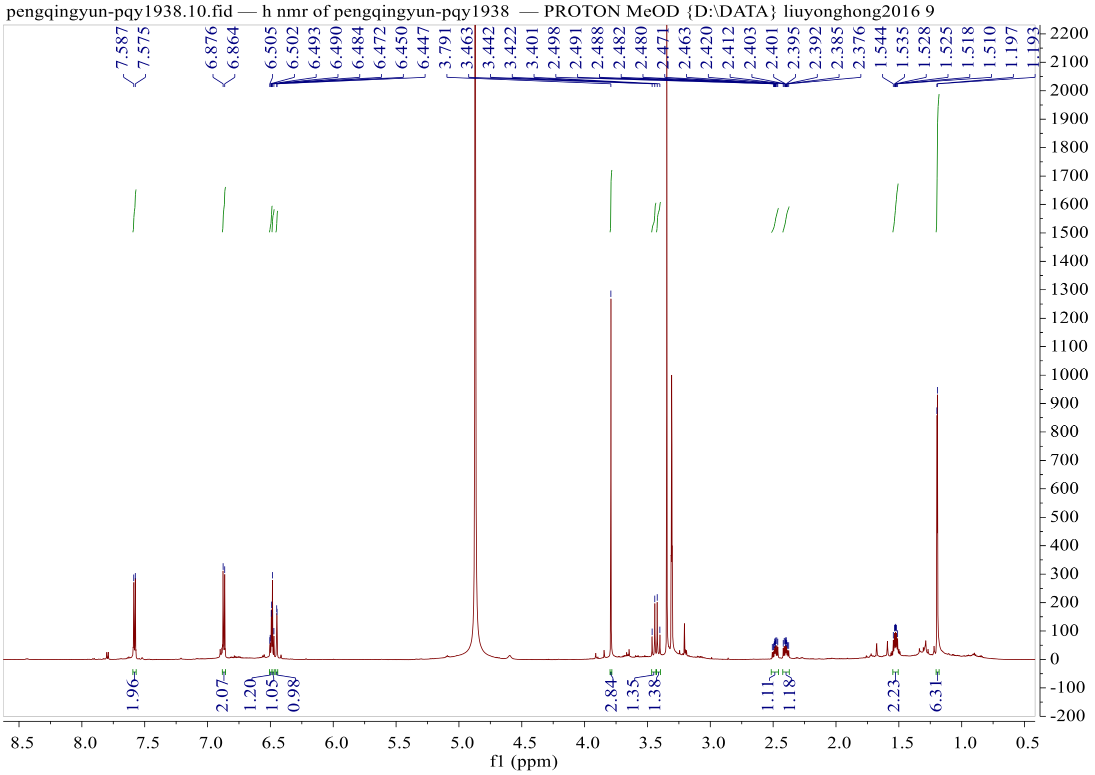

Figure S33. ^{1}H NMR spectrum of **11** (CD_3OD, 700 MHz).

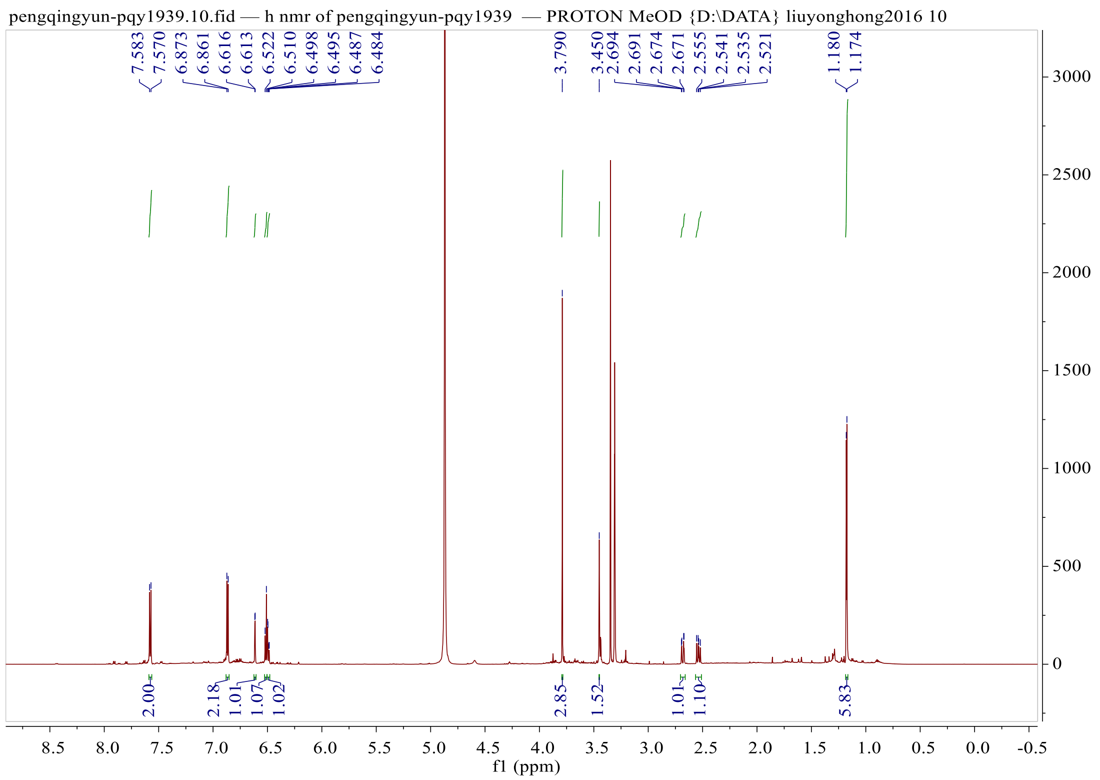

Figure S34. ^{1}H NMR spectrum of **12** (CD_3OD, 700 MHz).

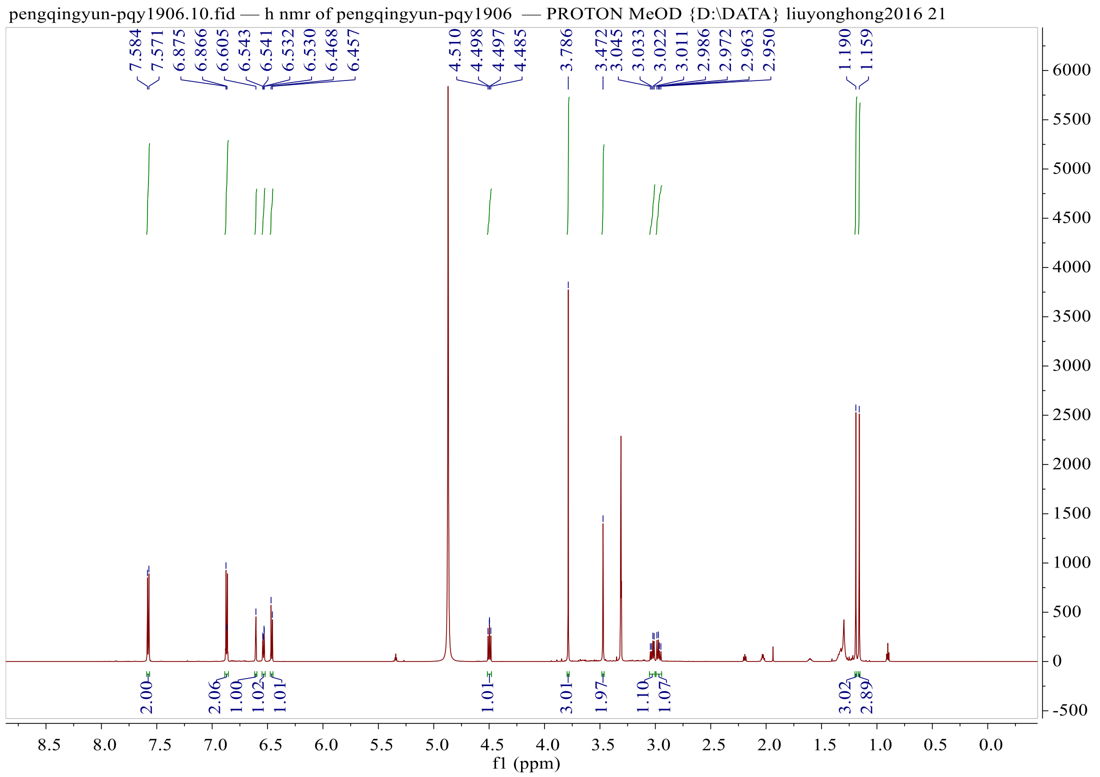

Figure S35. ^{1}H NMR spectrum of **13** (CD_3OD, 700 MHz).

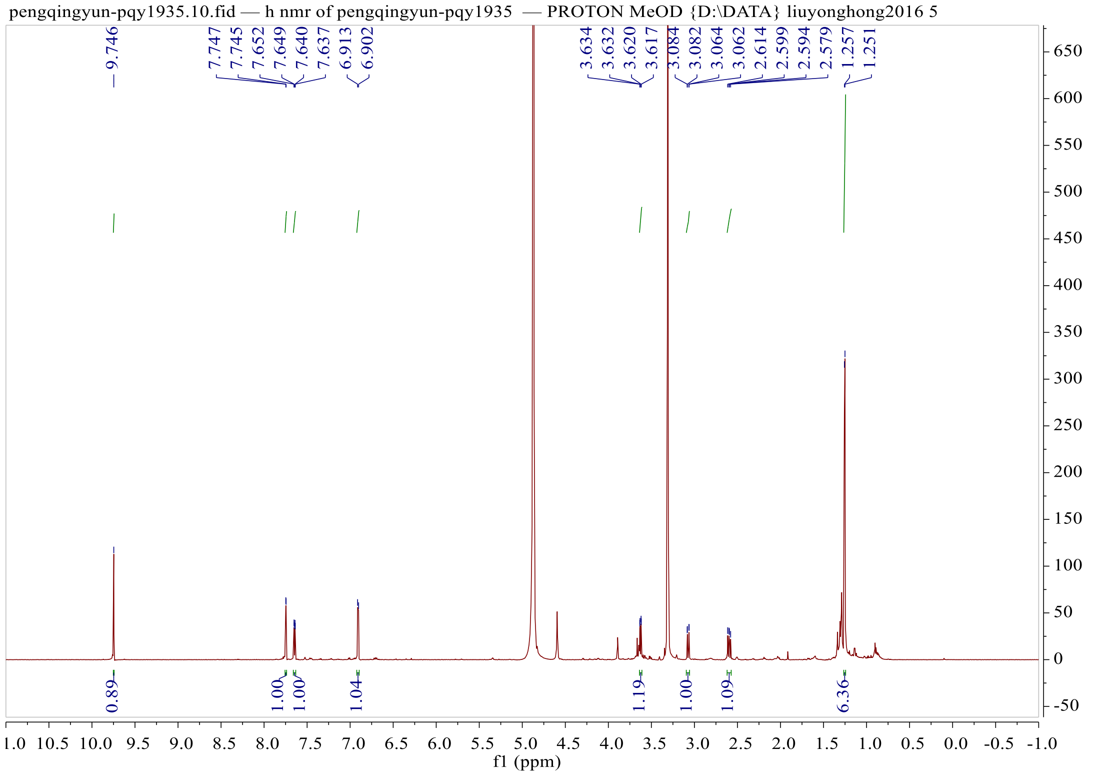

Figure S36. ^{1}H NMR spectrum of **14** (CD_3OD, 700 MHz).

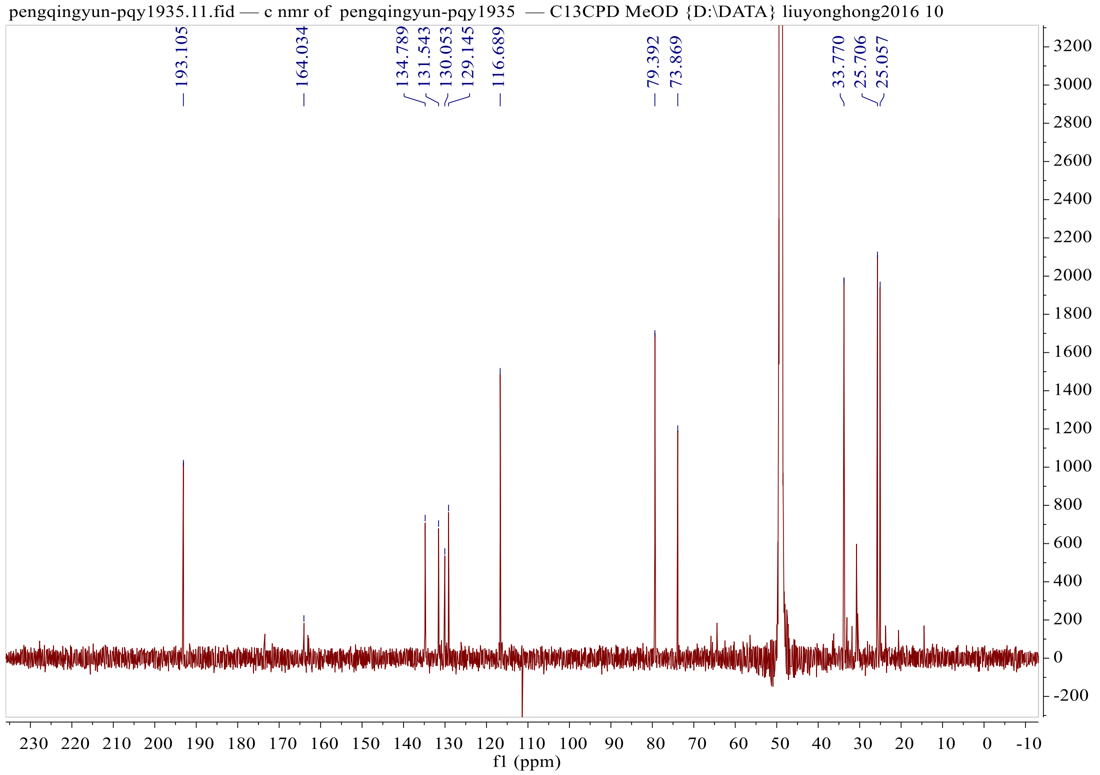

Figure S37. ^{13}C NMR spectrum of **14** (CD_3OD, 175 MHz).

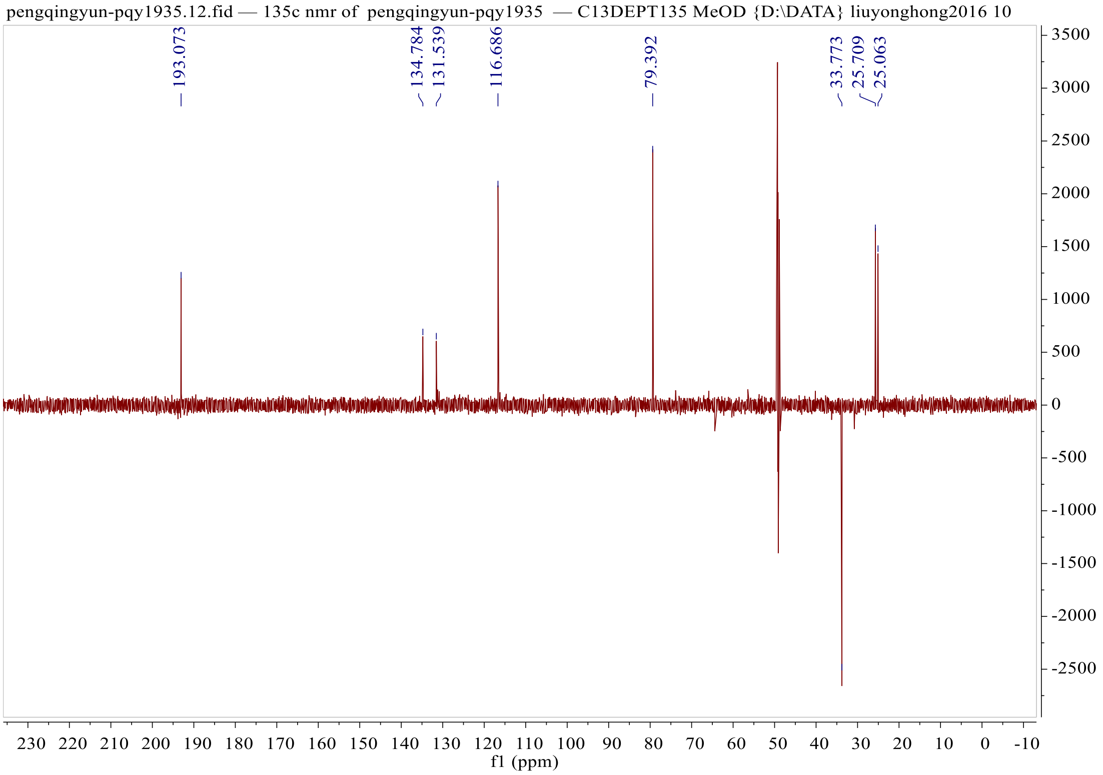

Figure S38. DEPT spectrum of **14** (CD_3OD).

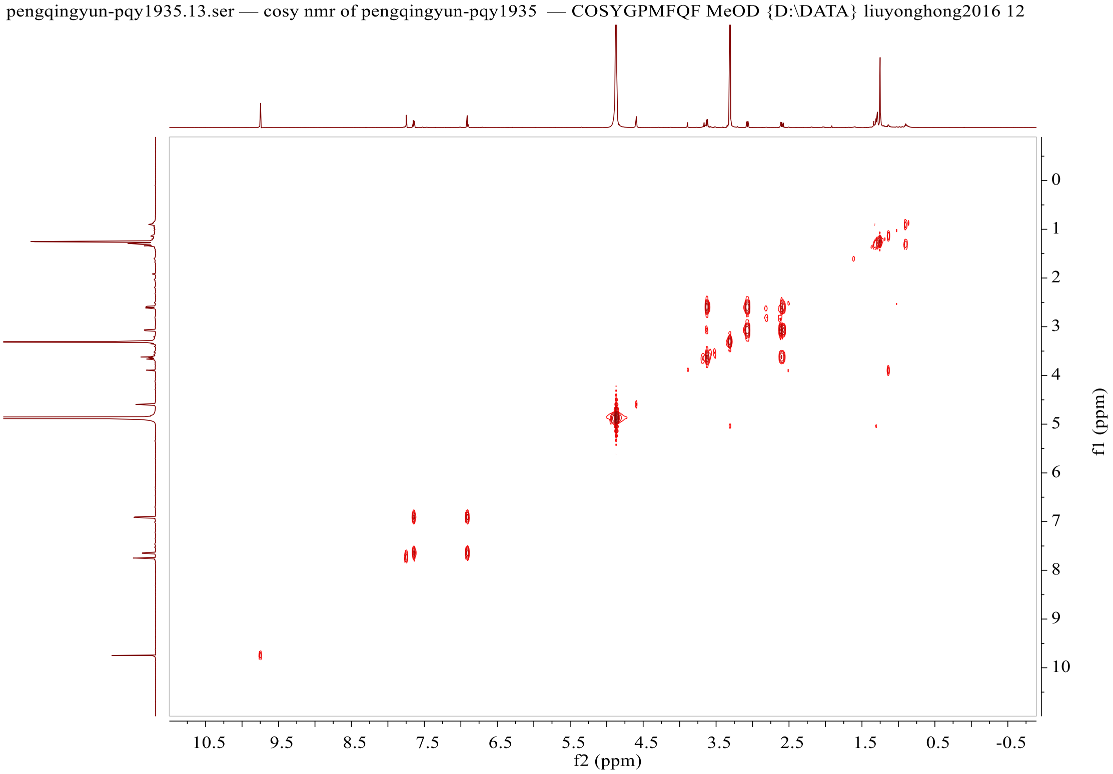

Figure S39. COSY spectrum of **14** (CD_3OD).

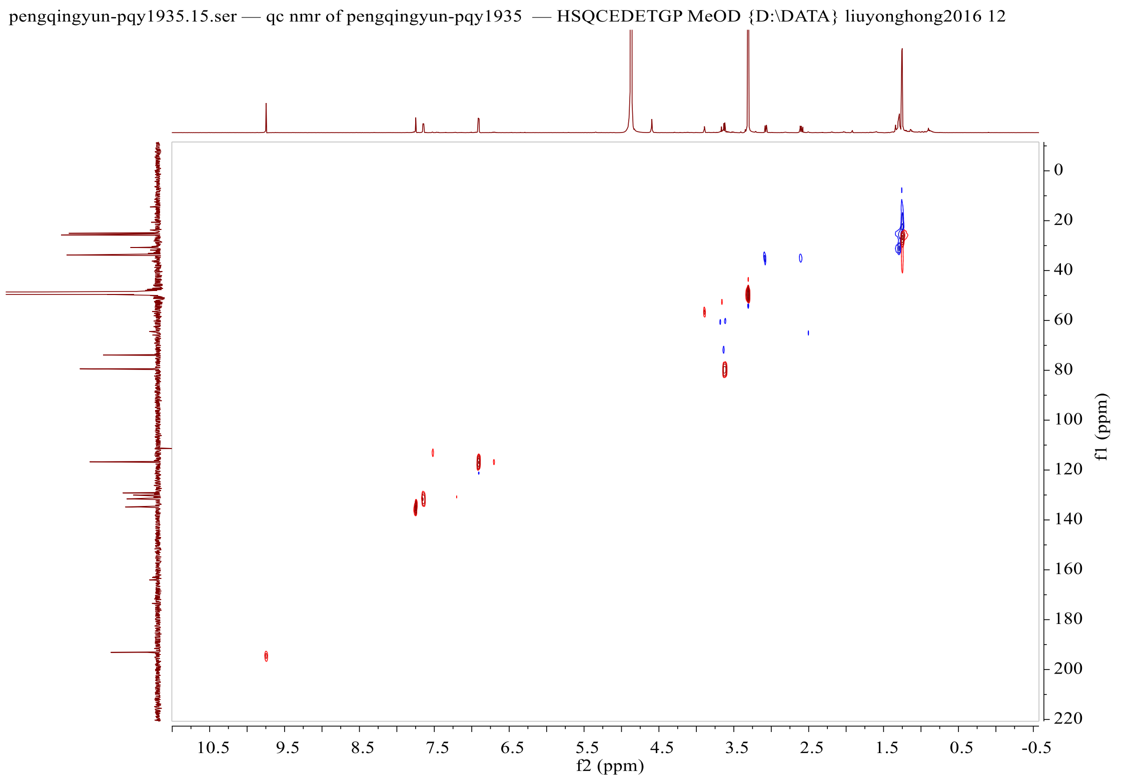

Figure S40. HSQC spectrum of **14** (CD_3OD).

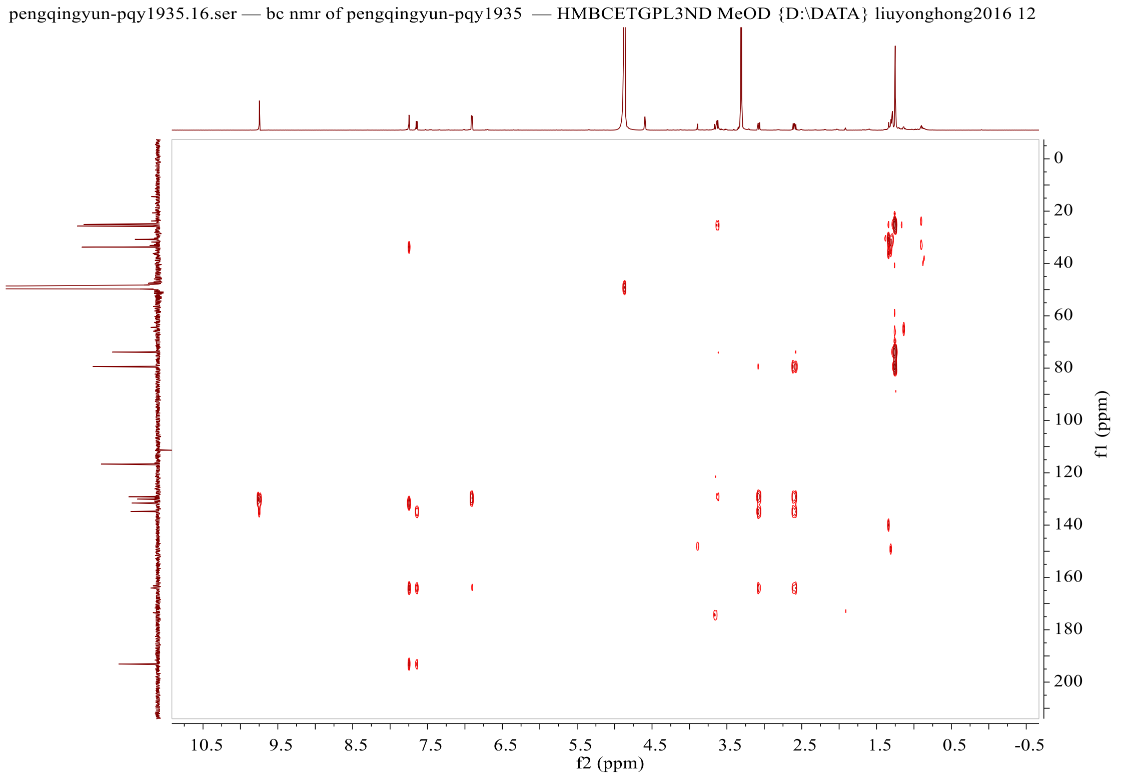

Figure S41. HMBC spectrum of **14** (CD_3OD).

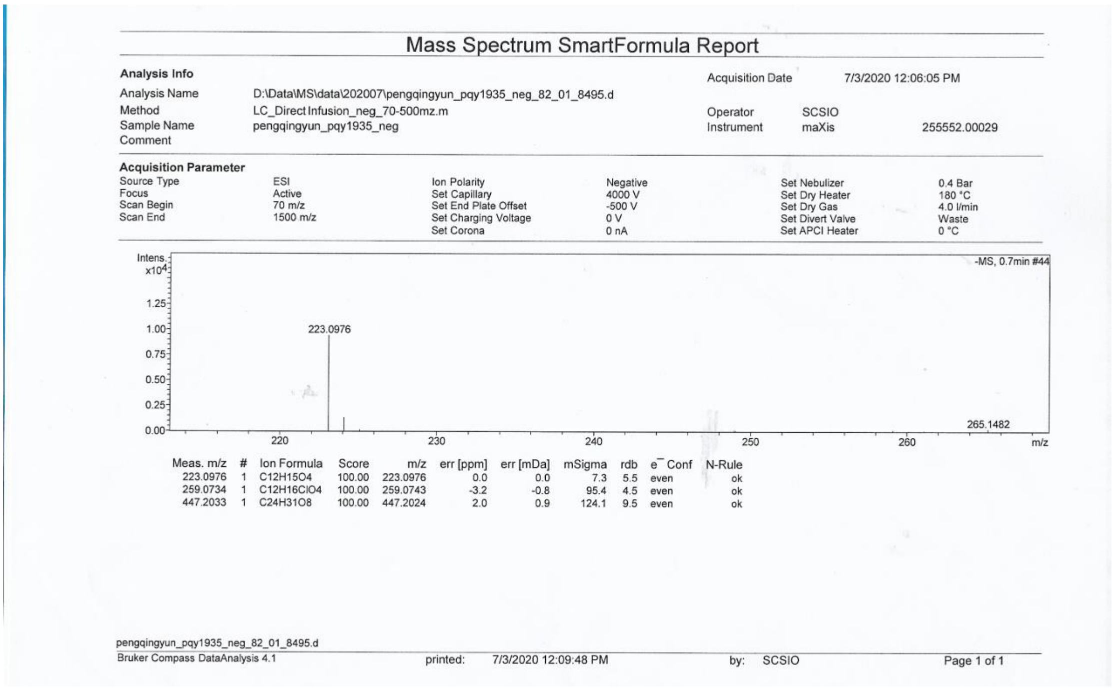
Mass Spectrum SmartFormula Report

Analysis Info

Analysis Name D:\Data\MS\data\202007\pengqingyun_pqy1935_neg_82_01_8495.d
Method LC_Direct Infusion_neg_70-500mz.m
Sample Name pengqingyun_pqy1935_neg
Comment

Acquisition Date 7/3/2020 12:06:05 PM
Operator SCSIO
Instrument maXis 255552.00029

Acquisition Parameter

Source Type	ESI	Ion Polarity	Negative	Set Nebulizer	0.4 Bar
Focus	Active	Set Capillary	4000 V	Set Dry Heater	180 °C
Scan Begin	70 m/z	Set End Plate Offset	-500 V	Set Dry Gas	4.0 l/min
Scan End	1500 m/z	Set Charging Voltage	0 V	Set Divert Valve	Waste
		Set Corona	0 nA	Set APCI Heater	0 °C

Meas. m/z	#	Ion Formula	Score	m/z	err [ppm]	err [mDa]	mSigma	rdb	e⁻ Conf	N-Rule
223.0976	1	C12H15O4	100.00	223.0976	0.0	0.0	7.3	5.5	even	ok
259.0734	1	C12H16ClO4	100.00	259.0743	-3.2	-0.8	95.4	4.5	even	ok
447.2033	1	C24H31O8	100.00	447.2024	2.0	0.9	124.1	9.5	even	ok

pengqingyun_pqy1935_neg_82_01_8495.d
Bruker Compass DataAnalysis 4.1 printed: 7/3/2020 12:09:48 PM by: SCSIO Page 1 of 1

Figure S42. HRESIMS spectrum of **14**.

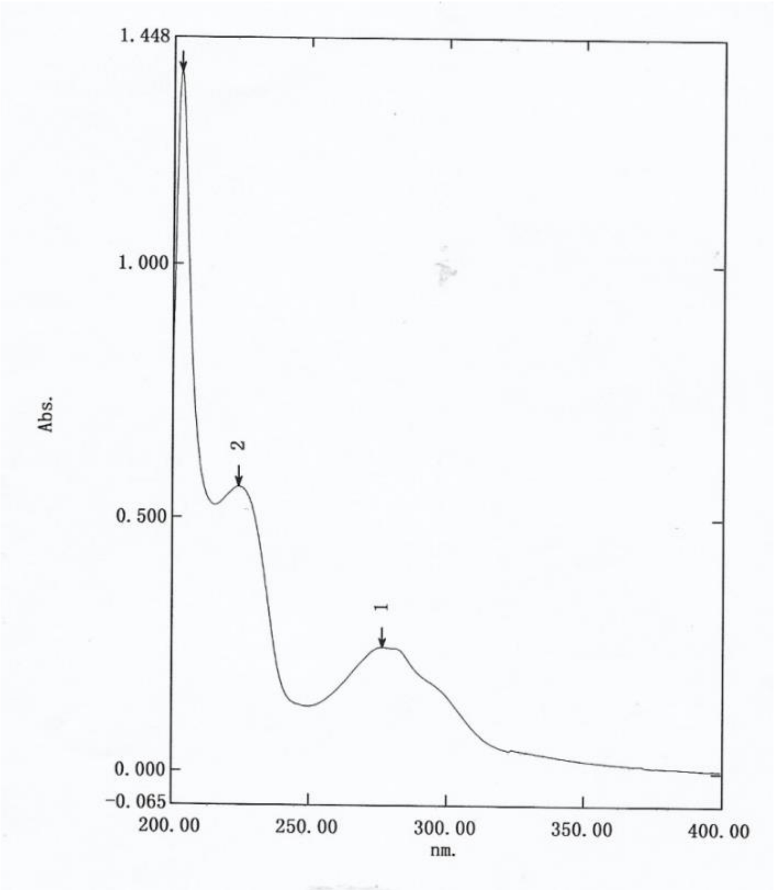

Figure S43. UV spectrum of **14**.
